# From reaction kinetics to dementia: A simple dimer model of Alzheimer’s disease etiology

**DOI:** 10.1371/journal.pcbi.1009114

**Published:** 2021-07-19

**Authors:** Michael R. Lindstrom, Manuel B. Chavez, Elijah A. Gross-Sable, Eric Y. Hayden, David B. Teplow

**Affiliations:** 1 Department of Mathematics, University of California, Los Angeles, California, United States of America; 2 Department of Neurology, David Geffen School of Medicine at the University of California, Los Angeles, California, United States of America; 3 Molecular Biology Institute and Brain Research Institute, University of California, Los Angeles, California, United States of America; SUNY Downstate MC, UNITED STATES

## Abstract

Oligomers of the amyloid *β*-protein (A*β*) have been implicated in the pathogenesis of Alzheimer’s disease (AD) through their toxicity towards neurons. Understanding the process of oligomerization may contribute to the development of therapeutic agents, but this has been difficult due to the complexity of oligomerization and the metastability of the oligomers thus formed. To understand the kinetics of oligomer formation, and how that relates to the progression of AD, we developed models of the oligomerization process. Here, we use experimental data from cell viability assays and proxies for rate constants involved in monomer-dimer-trimer kinetics to develop a simple mathematical model linking A*β* assembly to oligomer-induced neuronal degeneration. This model recapitulates the rapid growth of disease incidence with age. It does so through incorporation of age-dependent changes in rates of A*β* monomer production and elimination. The model also describes clinical progression in genetic forms of AD (e.g., Down’s syndrome), changes in hippocampal volume, AD risk after traumatic brain injury, and spatial spreading of the disease due to foci in which A*β* production is elevated. Continued incorporation of clinical and basic science data into the current model will make it an increasingly relevant model system for doing theoretical calculations that are not feasible in biological systems. In addition, terms in the model that have particularly large effects are likely to be especially useful therapeutic targets.

## Author summary

Oligomeric assemblies of A*β* are hypothesized to be seminal pathologic agents in Alzheimer’s disease (AD). Mechanistic studies of oligomerization and neurotoxicity in humans are currently impossible, yet such studies promise to advance efforts toward target identification and drug development. To overcome this hurdle, we developed a simple, mathematical model parameterized using experimental data extant. The model couples the kinetics of oligomerization with oligomer toxicity and enables determination of age-related changes in AD risk and hippocampal volume, the effects of traumatic brain injury on lifetime AD risk, gene dosage effects, and the effects of spatial variation in A*β* monomer concentrations on millimeter scales. The model is easily interpretable and provides a foundation for development of more comprehensive models of AD development and progression.

## Introduction

Alzheimer’s disease (AD) currently is the 6th leading cause of death in the U.S. and its prevalence continues increasing rapidly [1]. Thus, there is a critical need for the development of effective preventive, ameliorative, or curative therapeutics. Unfortunately, none exist [2]. This is due in part to the multifactorial nature of AD, which makes contemporaneous study of the entire system infeasible and requires researchers to focus on smaller system elements. One such element is amyloid plaque formation. Plaques in the brains of those with AD are extracellular deposits of long protein fibrils formed by the amyloid *β*-protein (A*β*) and one of the pathognomonic features of AD. This inspired the hypothesis that fibril formation is the seminal pathologic event in AD [3]. However, this “amyloid cascade hypothesis,” has largely been supplanted by the “oligomer cascade hypothesis,” which suggests that pre-fibrillar structures, oligomers, are the most important toxic agents [4]. Like AD itself, the process by which monomeric A*β* forms fibrils is complicated and involves a multitude of small, oligomeric assemblies, as well as large, pre-fibrillar precursor structures [5]. It has been suggested that oligomers as small as dimers may be the most important of these assemblies [6]. It also is important to note that A*β* is but one player in AD etiology. The roles of tau, inflammation, mitochondrial dysfunction, etc. remain to be incorporated into a single unifying hypothesis.

A*β* oligomerization and its effect on neurons in vitro and in vivo are being studied intensively (for recent reviews, see [7, 8]). Studies in our group [9] and others [10–12] have focused on the structural biology and kinetics of oligomerization and fibril formation. These studies have sought to relate these biophysical aspects of A*β* assembly to disease occurrence, pathology, and progression. Unfortunately, little is known about the relationship of oligomer states to the development of disease. It is clear that the constitutive level of A*β* production correlates directly with time of onset and severity of disease. This is quite apparent in people with Down’s syndrome, who possess three copies of the amyloid precursor protein gene that encodes A*β* and who tend to develop AD early in life, with some showing symptoms as early as age 40 [13]. Simple gene dosage extrapolation suggests that amyloid precursor protein (APP) concentration should be 150% the level found in normal individuals. This indeed has been the case in humans [14, 15]. In addition, studies by Cheon et al. [16] have shown that immunoreactive APP species are expressed in Down’s syndrome brains at even higher levels (∼1.8–2.7-fold) than is A*β*. Higher A*β* expression also is observed in rare familial forms of AD that are characterized by mutations in APP or the enzymes responsible for its production [17]. These mutations result in increased concentrations of A*β* or an increase in the relative amount of two forms of the protein, A*β*40 and A*β*42. A*β*42 is only two amino acids longer (42 vs. 40) than A*β*40, yet its pathogenicity is substantially higher.

Though A*β* is produced in the brain throughout life, AD is not usually observed before age 65 [18]. AD risk increases exponentially after that, reaching approximately 30% by age 85 [19]. Age is the most important risk factor for sporadic AD [20], but genetics also play a role. Apolipoprotein E, which can exist in the body in three different forms—ApoE2, ApoE3, and ApoE4—is a cholesterol carrier protein. The type of apolipoprotein E one expresses also has a significant effect on risk [21] and risk is increased substantially in individuals that express ApoE4 [21]. Blunt force trauma to the head, e.g., traumatic brain injury (TBI) or chronic traumatic encephalopathy (CTE), now are understood to be significant risk factors as well [22, 23].

Here, we report the creation of a mathematical model of the time-dependence of AD progression and its relationship to the kinetics of A*β* production, elimination, and toxicity. The model unites dynamic processes occurring at the protein level (A*β* oligomerization) and between A*β* and neurons, processes of hippocampal atrophy, and clinical disease development over the life span of the individual. The model predicts biologically significant time scales for development of AD; offers explanations for how blunt force trauma, Down’s syndrome, and changes in hippocampal volume affect disease risk; illustrates how individual rates of age-related neuronal degeneration affect disease prevalence and incidence; and provides mechanistic insight into how the disease may spread in the brain.

## Assumptions and model development

The oligomer cascade hypothesis posits that neuronal death in AD is primarily due to oligomers. In our model, we are therefore interested in describing the coupling between oligomers and loss in neuronal viability. This also requires modeling the kinetics of A*β* assembly. Our foci are on early stages of A*β* oligomerization under some simplifying assumptions, one of which is the division of A*β* peptide forms into monomers and two classes of oligomer, dimers and all others (which we refer to as higher-order oligomers (HOOs)). We examine how oligomerization correlates with AD development and progression.

**Fig 1:**
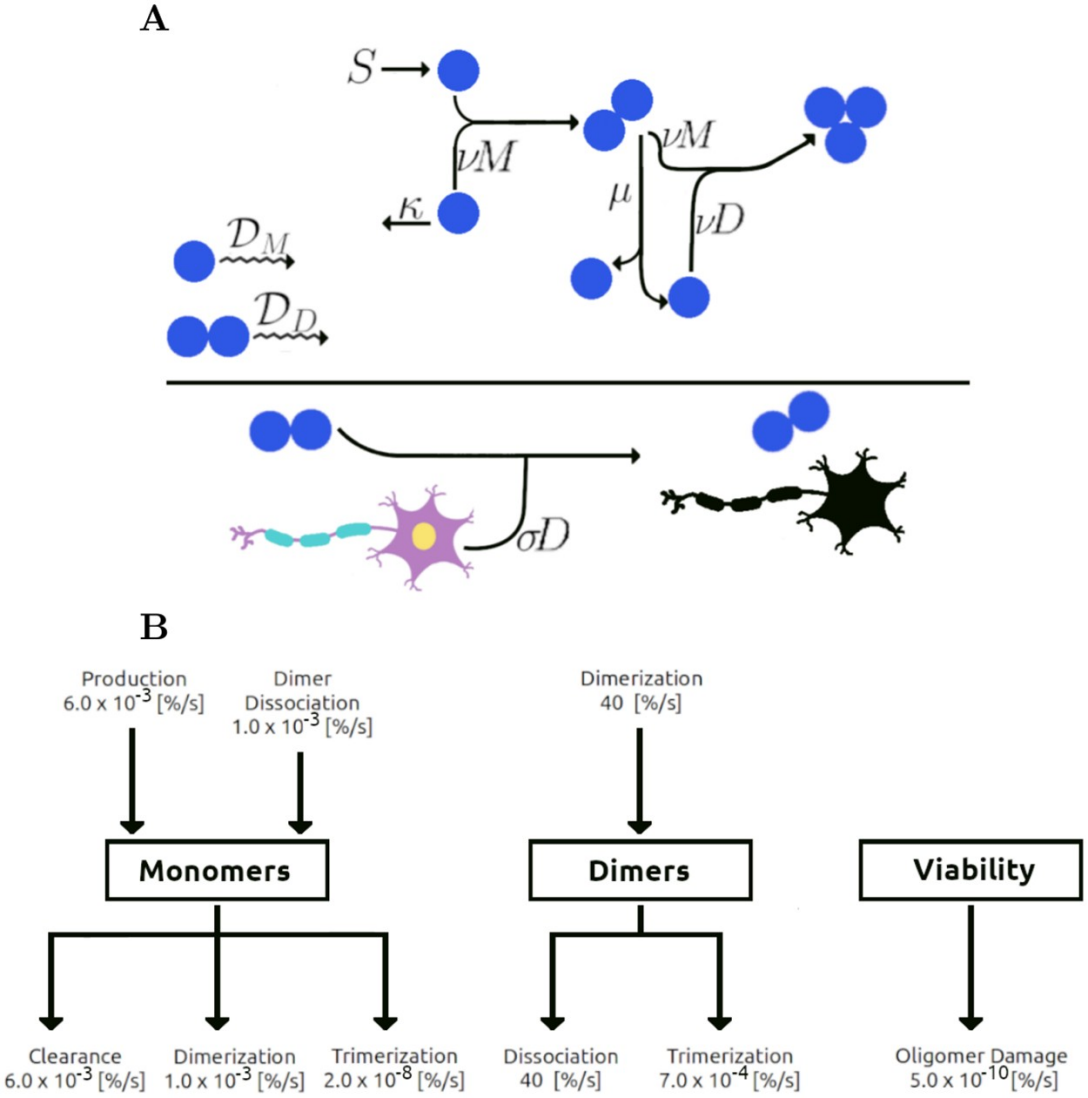
Model scheme. *A*: Monomers are produced at rate *S* (zeroth order) and cleared at a rate *κ* (first order). Two monomers combine to form a dimer with rate constant *ν* (second order) and a dimer can dissociate at rate *μ* (first order) into two monomers. Monomers and dimers can combine to form trimers at rate *ν* (second order), with negligible backwards reactions. Neurons are killed at a rate *σ* times the dimer concentration. Thus, as the dimer concentration rises, so does the speed of neuronal death. Monomers and dimers diffuse with diffusivities $D_{M}$ and $D_{D}$, respectively. *B*: Representative production/loss rates of individual components when concentrations are at their baselines values in Table 1, without the rates changing with age. Incoming arrows represent gain/production; outgoing arrows represent loss/clearance. For example, in each second, 40% of the dimer concentration is lost due to dissociation (dimers have a very short lifespan) and gained from dimerization illustrating that the monomer-dimer equilibrium is fast relative to other equilibria, whereas cell viability is lost very slowly.

We begin by describing the concentration of monomers *M* and dimers *D* in the interstitial fluid. While HOOs may also be toxic, we show in the S1 Text (Mathematical Details section) that the HOOs play a negligible role in the model when compared to the dimers. Dimers have also been found to be the most abundant form of A*β* in the human brain [24] and they are toxic [4] to neurons. Strong evidence, to our knowledge, does not exist showing monomers are toxic, hence they are excluded as factors in the loss of viable cells. We model viable cell density *V* as being lost at a rate proportional to the dimer concentration with proportionality constant *σ*. At each instant, the percentage chance that one develops AD for each one percent decrease in viable neurons is defined to be *γ*, what we refer to as the *neuronal death elasticity of AD risk* (similar to the economic concept of “price elasticity of demand,” the percent change in demand for each percent increase in price [25]). For kinetics, we stipulate that monomers are produced at a rate *S* and they are cleared at a rate *κ*; dimerization (and the formation of HOOs through monomer addition) occurs at a rate *ν*, with a dissociation rate *μ*. To consider the effects of diffusion, we assign monomers a diffusivity $D_{M}$ and dimers a diffusivity $D_{D}$. Lastly, there is evidence for rate constants such as *S* and *κ* being age dependent with *S* increasing with age and *κ* decreasing with age. When rate constant time-dependence is considered, we use linear models where $\lambda_{S}^{G}$ and $\lambda_{S}^{D}$ are the time it takes for *S* to double in the general and Down Syndrome populations, and λ_*κ*_ is the time when *κ* would reach 0 (at which point the model is no longer accurate). See Fig 1 for a schematic of the mechanisms.

We note that our own prior experimental data [5, 26], and recent work by Hasecke *et al*. [27], show that small oligomers, including trimers, exist in a rapid equilibrium with other assembly species. We have not modeled this entire equilibrium state because our model is predictive in nature, not accommodative, i.e., we do not want to fit our data to all data that exist *a priori*, which would constrain the model’s potential usefulness and the possibility of discovery. Instead, we chose a model-building strategy that incorporates only those elements that we predict will control the system. This means that some known elements have been omitted and others may not correspond to known factors influencing A*β* assembly.

Recently, Knowles et al. [28, 29] studied the molecular pathways of A*β*-42 oligomer and fibril formation, finding that oligomer dissociation is favored significantly over continued monomer addition leading to fibril formation (under their experimental conditions). Thus, the focus of our model upon low order oligomers is an important step in understanding AD etiology.

**Fig 2:**
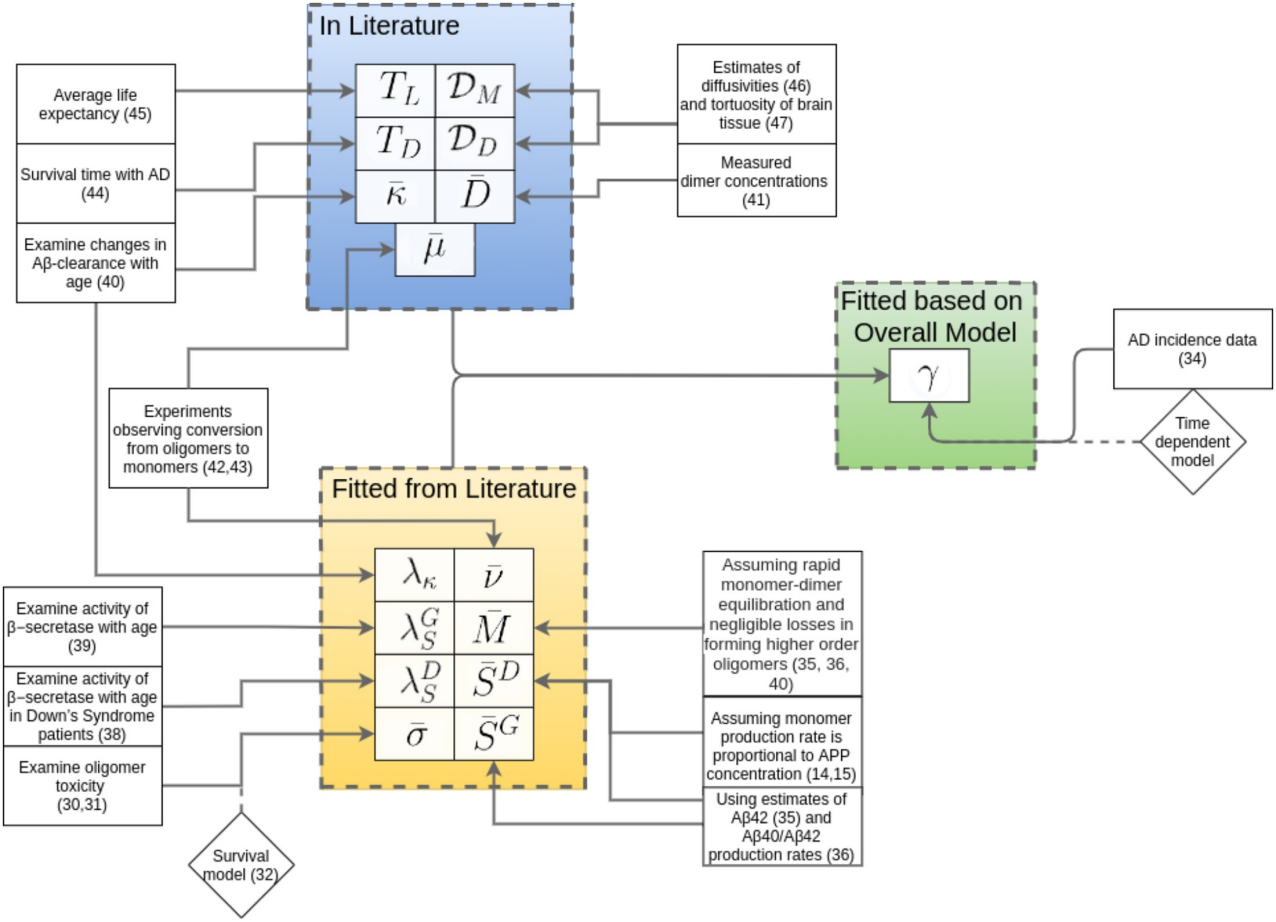
Means of obtaining model parameters. Some parameters (blue) were taken from published values in the literature; others (yellow-orange) were fitted based on experimental data; the value *γ* (green) is fitted from our overall model with reference to clinical data.

Full details of parameter estimation are provided in the S1 Text (Parameter Estimation section). Here, we provide an overview of the steps taken to arrive at the parameters in Table 1. See Fig 2 for an illustration. Note that a variable with a bar indicates a representative scale/size for that variable. For instance, monomer clearance *κ* could be time-dependent and $\bar{\kappa }$ is a representative size of *κ*. From experiments with brain slice cultures and mixed neuron-glial cultures, oligomer toxicity was examined at different concentrations [30, 31], allowing us to estimate $\bar{\sigma }$ from a survival model [32]. The loss of neurons is coupled with increased risk of AD through the neuronal death elasticity of AD risk *γ*, based on the notion that AD develops when one or more neurons [33] critical for memory processes dies. We estimate *γ* from AD incidence data and our model [34].

**Table 1:** Values of parameters within the model. See Fig 2 for an explanation of how these values were determined and their sources; Bars (e.g., $\bar{S}$), indicate a quantity representative of that in a healthy brain; *Lit*. denotes values listed in literature; *Inf*. denotes values inferred or fit from published data; *Mod*. indicates a value fit from our model with reference to literature; *Def*. denotes the definition of a value used in our study in various calculations, derived from other quantities in the table.

Parameter	Meaning	Value	Source
$\bar{\kappa }$	Baseline monomer loss rate	6.15 × 10^−5^ s^−1^	Lit. [40]
$\bar{D}$	Characteristic dimer concentration	1.00 × 10^−12^ M	Lit. [41]
$\bar{\mu }$	Baseline dimer dissociation rate	0.400 M^−1^ s^−1^	Lit. [42, 43]
***T*_*D*_**	Survival time after AD diagnosis	2.23 × 10^9^ s [7.1 yr]	Lit. [44]
***T*_*L*_**	Life expectancy in United States	2.48 × 10^9^ s [78.5 yr]	Lit. [45]
$D_{M}$	Monomer diffusivity	5.47 × 10^−7^ cm^2^ s^−1^	Lit. [46, 47]
$D_{D}$	Dimer diffusivity	4.30 × 10^−7^ cm^2^ s^−1^	Lit. [46, 47]
${\bar{S}}^{G}$	Baseline monomer production rate (general)	3.63 × 10^−12^ M s^−1^	Inf. [35, 36]
${\bar{S}}^{D}$	Baseline monomer production rate (Down Syndrome)	5.45 × 10^−12^ M s^−1^	Inf. [14, 15, 35, 36]
$\bar{M}$	Characteristic monomer concentration	5.90 × 10^−8^ M	Inf. [35, 36, 40]
$\bar{\nu }$	Baseline monomer combination rate	115 s^−1^	Inf. [42, 43]
$\bar{\sigma }$	Baseline cell-dimer damage rate	4.94 M^−1^ s^−1^	Inf. [30–32]
$\lambda_{S}^{G}$	Linear growth doubling time of production in general population	4.85 × 10^9^ s [154 yr]	Inf. [39]
$\lambda_{S}^{D}$	Linear growth doubling time of production in Down Syndrome population	2.72 × 10^9^ s [86.2 yr]	Inf. [38]
**λ_*κ*_**	Linear decay time to zero for clearance	3.60 × 10^9^ s [114 yr]	Inf. [40]
***γ***	Neuronal death elasticity of AD risk	6.01 × 10^−1^	Mod. [34]
$\bar{x}$	Characteristic lengthscale	9.43 × 10^−2^ cm	Def.
$\bar{t}$	Characteristic timescale	1.62 × 10^4^ s [4.5 hr]	Def.
$\bar{U}$	Characteristic neuronal damage rate	4.94 × 10^−12^ s^−1^ [1.56 × 10^−4^ yr^−1^]	Def.
$\bar{\omega }$	Characteristic AD development rate	2.96 × 10^−12^ s^−1^ [9.34 × 10^−5^ yr^−1^]	Def.

Through estimates of A*β*42 monomer production rates [35] and the ratio of A*β*42 to A*β*40 in the brain [36], we estimate ${\bar{S}}^{G}$. We estimate ${\bar{S}}^{D}$ by assuming the production rate is proportional to APP concentration which is increased by 50% [14, 15]. Further studies that show how the activity of *β*-secretase may increase with age [37–39] allow us to estimate $\lambda_{S}^{G}$ and $\lambda_{S}^{D}$. Likewise, experiments that show how A*β* clearance changes with age [40] allow us to estimate $\bar{\kappa }$ and λ_*κ*_. Measured soluble A*β* concentrations from the literature allow us to estimate the characteristic scale of the dimer concentration [41]. Based on some further assumptions relating to the relative speed of the different reactions and experiments on relevant rate constants [42, 43] we arrive at estimates for the characteristic scale of the monomer concentration, the dissociation rate $\bar{\mu }$, and dimerization rate $\bar{\nu }$. In modeling prevalence, we consider the number of years a patient survives with AD. As a simplification, we assume that after a course of illness of length *T*_*D*_ = 7.1 years, all AD patients die [44]. We also estimate lifetime risk by using the average life expectancy in the United States of *T*_*L*_ = 78.5 years [45].

### Monomers and dimers

We denote *t* as time (age) so that *t* = 0 corresponds to birth, and *M* and *D* as the concentrations of monomers and dimers, respectively. The kinetics are modelled by the partial differential equations (PDEs) Eqs (1) and (2).

$$\begin{array}{cc} \frac{\partial M}{\partial t} & =D_{M}\Delta M+S-\kappa M-2\nu M^{2}-\nu MD+2\mu D \end{array}$$ (1)

$$\begin{array}{cc} \frac{\partial D}{\partial t} & =D_{D}\Delta D+\nu M^{2}-\mu D-\nu MD \end{array}$$ (2)

Including HOOs is possible. We provide heuristics of what this could look like in the S1 Text (Mathematical Details section) but also show that the concentrations and toxicity effects of these HOOs are negligible based on our parameter estimates. Thus, Eqs (1) and (2) suppress terms of negligible size.

If only time dynamics are considered without modeling space, Eqs (1) and (2) are replaced by ordinary differential equations (ODEs):

$$\begin{array}{cc} \frac{\text{d}M}{\text{d}t} & =S-\kappa M-2\nu M^{2}-\nu MD+2\mu D \end{array}$$ (3)

$$\begin{array}{cc} \frac{\text{d}D}{\text{d}t} & =\nu M^{2}-\mu D-\nu MD \end{array}$$ (4)

It has been observed that A*β* clearance rate decreases with age [40], whereas the activity of *β*-secretase increases [37]. Many forms could be chosen for *S*(*t*) and *κ*(*t*) for these respective rates at a time *t*. For *S*(*t*) and *κ*(*t*), we *employ linear models, to be as simple as possible*, using data presented in the S1 Text (Parameter Estimation section) to write

$$\begin{array}{c} S\left(t\right)=\bar{S}\left(1+t/\lambda_{S}\right),\;\kappa \left(t\right)=\bar{\kappa }\left(1-t/\lambda_{\kappa }\right), \end{array}$$ (5)

where

$$\begin{array}{c} \left(\bar{S},\lambda_{S}\right)=\{\begin{array}{c} ({\bar{S}}^{G},\lambda_{S}^{G})\;\;\text{in}\;\text{the}\;\text{general}\;\text{population}\\ ({\bar{S}}^{D},\lambda_{S}^{D})\;\;\text{in}\;\text{the}\;\text{Down}\;\text{Syndrome}\;\text{population} \end{array} \end{array}$$ (6)

depend on whether an individual is in the general or Down Syndrome populations. Note that individual equations on lines containing multiple equations are referenced with a subscript indicating their order in the line, e.g., the equation defining *S*(*t*) would be referred to by (5)_1_. The form of *S*(*t*) assumes that monomer production is directly proportional to the activity of *β*-secretase and to the concentration of APP. It is possible that a combination of genetics and lifestyle factors play a role and may modify the rates that *S* and *κ* change. It also is possible that *σ*, *ν*, and *μ* vary with age. However, literature extant does not provide sufficient insights to model this quantitatively, thus we treated these rates as constants. We note that the model loses validity for *t* near 114 yr as the clearance rate reaches zero around this point. From the values of $\lambda_{S}^{G}$, $\lambda_{S}^{D}$, and λ_*κ*_, *S*(*t*) and *κ*(*t*) change very slowly.

### Cell viability, incidence, and prevalence

Over each small volume of brain, we model the cell viability 0 ≤ *V* ≤ 1 as the number density (number per unit volume) of viable neuronal cells divided by the number density of neurons in perfectly healthy brain tissue. We model the decrease in this viability with a hazard function which is proportional to the oligomer concentration by

$$\begin{array}{cc} \frac{\partial V}{\partial t} & =-\sigma DV. \end{array}$$ (7)

Viability decreases faster the more oligomers are present; *σ* is a coupling constant for oligomer toxicity. We fit for *σ* [48] using cell viability assay data [30, 31]. We note the model could easily be generalized to accommodate other potentially toxic proteins, e.g., tau, by adding additional damage terms to Eq (7) and incorporating an equation describing the concentration of those proteins.

As described in the S1 Text (Parameter Estimation section), given the viability model with homogeneous brain tissue, we also identify the survivorship function *H*(*t*) (fraction of individuals who do not have AD by age *t*), incidence *I*(*t*) (per capita rate of AD development of age *t* individuals), prevalence *P*(*t*) (fraction of individuals age *t* with AD), and Υ(*t*_1_, *t*_2_) (cumulative risk of AD between ages *t*_1_ and *t*_2_, with no AD up to *t*_1_) through

$$\begin{array}{cccc} H(t) & =V^{\gamma } & & I(t)=\gamma \sigma D \end{array}$$ (8)

$$\begin{array}{cccc} P(t) & =1-{\text{e}}^{-\int_{\text{max}\{0,t-T_{D}\}}^{t}I\left(s\right)\text{d}s} & & ϒ\left(t_{1},t_{2}\right)=1-{\text{e}}^{-\int_{t_{1}}^{t_{2}}I\left(s\right)\text{d}s} \end{array}$$ (9)

where Eqs (8) and (9) are valid for 114 yr = λ_*κ*_ > *t*_2_, *t*_1_. The choice of *γ* in Table 1 is made by considering incidence data.

### Solutions

Solving these equations can be complicated. However, in the parameter regime considered, various approximations are possible owing to a separation of time scales. There are fast time scales for dimer dissociation (∼ms); intermediate time scales for monomer decay (∼h); and long time scales for changes in kinetic rate constants and loss of neuronal health (decades). The relative sizes of terms can also be exploited. Since the losses due to trimerization are, by model construction, negligible with respect to dimer evolution, the dimer concentration is controlled by dimerization and dimer dissociation, which forces *D* to scale quadratically with *M*. This also means the monomer concentration is described through a balance of production, clearance, and possibly diffusion, which can also be solved analytically. Finally, owing to the slow changes in rate constants, monomers and dimers are always quasi-static. Combining this with the slow change in neuronal viability due to dimers at their natural concentrations makes the simple first order decay of cell viability *V* with respect to dimer concentration straightforward to solve over the long time scales of AD development.

#### Ordinary differential equations

With only time-dependence (assuming conditions in the brain are uniform throughout), after the effects of initial conditions are no longer relevant (see S1 Text (Mathematical Details section)), we have

$$\begin{array}{c} M\left(t\right)=\frac{S(t)}{\kappa (t)},\;D\left(t\right)=\frac{\nu \left(t\right)S^{2}\left(t\right)}{\mu \left(t\right)\kappa^{2}\left(t\right)},\;V\left(t\right)=\text{exp}\left(-\int_{0}^{t}U\left(u\right)\text{d}u\right) \end{array}$$ (10)

where we define

$$\begin{array}{c} U\left(t\right)=\frac{\sigma \left(t\right)\nu \left(t\right)S^{2}\left(t\right)}{\mu \left(t\right)\kappa^{2}\left(t\right)},\;\omega \left(t\right)=\gamma U\left(t\right). \end{array}$$ (11)

From (10)_2_, we obtain the incidence, prevalence, and lifetime risk of the disease with Eqs (8)_2_ and (9)_1−2_. In the special case that *S*, *κ*, *μ*, *ν* and *σ* are constant, representing ideal aging whereby production, clearance, and other rates are optimal throughout life, we have

$$\begin{array}{cccccc} M & =\frac{\bar{S}}{\bar{\kappa }} & & D=\frac{\bar{\nu }{\bar{S}}^{2}}{\bar{\mu }{\bar{\kappa }}^{2}} & & V\left(t\right)=\text{exp}\left(-\bar{U}t\right) \end{array}$$ (12)

$$\begin{array}{cccc} H(t) & =\text{exp}(-\bar{\omega }Ut) & & I\left(t\right)=\bar{\omega } \end{array}$$ (13)

$$\begin{array}{cccc} P(t) & =1-{\text{e}}^{-\bar{\omega }\text{min}\left\{t,T_{D}\right\}} & & ϒ\left(t_{1},t_{2}\right)=1-{\text{e}}^{-\bar{\omega }\left(t_{2}-t_{1}\right)}, \end{array}$$ (14)

where

$$\begin{array}{c} \bar{U}=\frac{\bar{\sigma }\bar{\nu }{\bar{S}}^{2}}{\bar{\mu }{\bar{\kappa }}^{2}},\;\bar{\omega }=\gamma \bar{U}. \end{array}$$ (15)

The value $\bar{U}$ is an estimate for the rate neurons die in perfectly healthy brain tissue. The value $\bar{\omega }$ is an estimate for the rate at which AD develops in the perfectly healthy population. Effectively, $\bar{U}$ and *U*(*t*) describe events occurring at the cellular level and $\bar{\omega }$ and *ω*(*t*) describe events at the population level.

#### Partial differential equations

To study spatial effects, we consider the question of a localized increase in A*β* monomer production and how this affects cells in the vicinity. We consider a *spherically symmetric* source of excess monomers. We consider a hypothetical scenario with $\kappa =\bar{\kappa },\mu =\bar{\mu },\nu =\bar{\nu }$, and $\sigma =\bar{\sigma }$. We choose $S=\bar{S}$ except over a sphere of radius $X^{*}=2\bar{x}$ centered at *x* = 0 where the monomer production is increased by *ρ* = 23.1%. There, $S=\bar{S}\left(1+\rho \right)$. The choice of X* is made so as to be on the order of $\bar{x}$, a characteristic length a monomer may diffuse before its clearance; and the choice of *ρ* comes from our findings on traumatic brain injury where $0.231\bar{S}$ is a representative increase in monomer production. We wish to study how the A*β* assemblies vary in space and how the viability changes over space and time. The solutions are presented in Eqs (16) and (17)_1−2_.

$$\begin{array}{cc} M(x) & =\frac{\bar{S}}{\bar{\kappa }}+\rho \frac{\bar{S}}{\bar{\kappa }}\{\begin{array}{c} \frac{{\text{e}}^{-\sqrt{\frac{\bar{\kappa }}{D_{M}}}\left|x\right|}}{\sqrt{\bar{\kappa }/D_{M}}\left|x\right|}[\sqrt{\frac{\bar{\kappa }}{D_{M}}}|x|\text{cosh}(\sqrt{\frac{\bar{\kappa }}{D_{M}}}|x|)-\text{sinh}(\sqrt{\frac{\bar{\kappa }}{D_{M}}}\left|x|\right)]\\ +\frac{\text{sinh}(\sqrt{\frac{\bar{\kappa }}{D_{M}}}|x|)}{\sqrt{\bar{\kappa }/D_{M}}\left|x\right|}[\sqrt{\frac{\bar{\kappa }}{D_{M}}}(\left|x\right|{\text{e}}^{-\sqrt{\frac{\bar{\kappa }}{D_{M}}}\left|x\right|}-X^{*}{\text{e}}^{-\sqrt{\frac{\bar{\kappa }}{D_{M}}}X^{*}})\\ -{\text{e}}^{-\sqrt{\frac{\bar{\kappa }}{D_{M}}}X^{*}}+{\text{e}}^{-\sqrt{\frac{\bar{\kappa }}{D_{M}}}\left|x\right|}],\\ \;|x|<X^{*}\\ \frac{{\text{e}}^{-\sqrt{\frac{\bar{\kappa }}{D_{M}}}\left|x\right|}}{\sqrt{\bar{\kappa }/D_{M}}\left|x\right|}[\sqrt{\frac{\bar{\kappa }}{D_{M}}}X^{*}\text{cosh}\left(\sqrt{\frac{\bar{\kappa }}{D_{M}}}X^{*}\right)-\text{sinh}\left(\sqrt{\frac{\bar{\kappa }}{D_{M}}}X^{*}\right)],\;\left|x\right|\geq X^{*}, \end{array} \end{array}$$ (16)

$$\begin{array}{cc} D(x) & =\frac{\bar{\nu }M{\left(x\right)}^{2}}{\bar{\mu }}\;V\left(x,t\right)=\text{exp}\left(-\frac{\bar{\sigma }\bar{\nu }M{\left(x\right)}^{2}}{\bar{\mu }}t\right) \end{array}$$ (17)

## Model predictions

In this section we focus primarily upon the results. Commentary on the predictive power of the model is in the Discussion. Readers can access our code at https://bitbucket.org/3k1m/dimer_model_ad/src/master/.

From the model developed, a series of comparisons can be made between our model and clinical observations. In general, we can consider our ODE model in two forms:

- A **static model** in which all rate constants are constant over a lifetime. This represents an ideal situation in which age-related decreases in monomer clearance rate, increases in monomer production, etc., do not take place. In this model, neuronal damage is as slow as possible.
- A **dynamic model** in which the rate constants *S* and *κ* vary as in Eq (5)_1−2_. Here, through aging, the rate constants change in disadvantageous directions.

While we believe the dynamic model is more accurate, explicitly taking into account the aging process (*S* and *κ* change over time), it does not always lend itself to simple analysis. The static model, although less quantitatively accurate, is particularly useful for gleaning qualitative insights into parameters because its solutions are simple expressions.

Our PDE model, which takes into account spatial variations in the system, is done with the kinetic rate constants being constant in time.

### AD incidence, prevalence, and lifetime risk

#### Clinical data

Age is the single leading risk factor for developing AD. In the ODE model, we can compare the predicted incidence, prevalence, and lifetime risk from the model with the clinical data. We consider the incidence rate (per person) in the United States [34], the AD prevalence by age range in the World Health Organization region AMRO A [19], and estimates of lifetime risk. The lifetime risk at age 60 for males is 13.9% and for females is 20.1% [49]. Averaging the two, we estimate the lifetime risk of AD is 17% at age 60. We investigate these data with the ODE model.

#### Model

The incidence, prevalence, and lifetime risk described here are given by Eqs 8)_2_, (9)_1,2_, (13)_2_ and (14)_1,2_.

#### Static model (*κ*, *S* constant)

Over a lifetime, the incidence would be a constant, $\bar{I}$, given by $\bar{I}=\bar{\omega }=9.34\times 10^{-5}\;{\text{yr}}^{-1}.$ By Taylor expanding (14)_1_, the prevalence at each age ≥ *T*_*D*_ = 7.1 yr is approximately constant, $\bar{P}$, with value $\bar{P}=\bar{\omega }T_{D}=0.0663\%,$ where *γ* is chosen based on the dynamic model below. We can use (14)_2_ with *t*_1_ = 60 y and *t*_2_ = *T*_*L*_ to estimate the lifetime risk of 0.17%.

#### Dynamic model (*κ*, *S* time-dependent)

**Fig 3:**
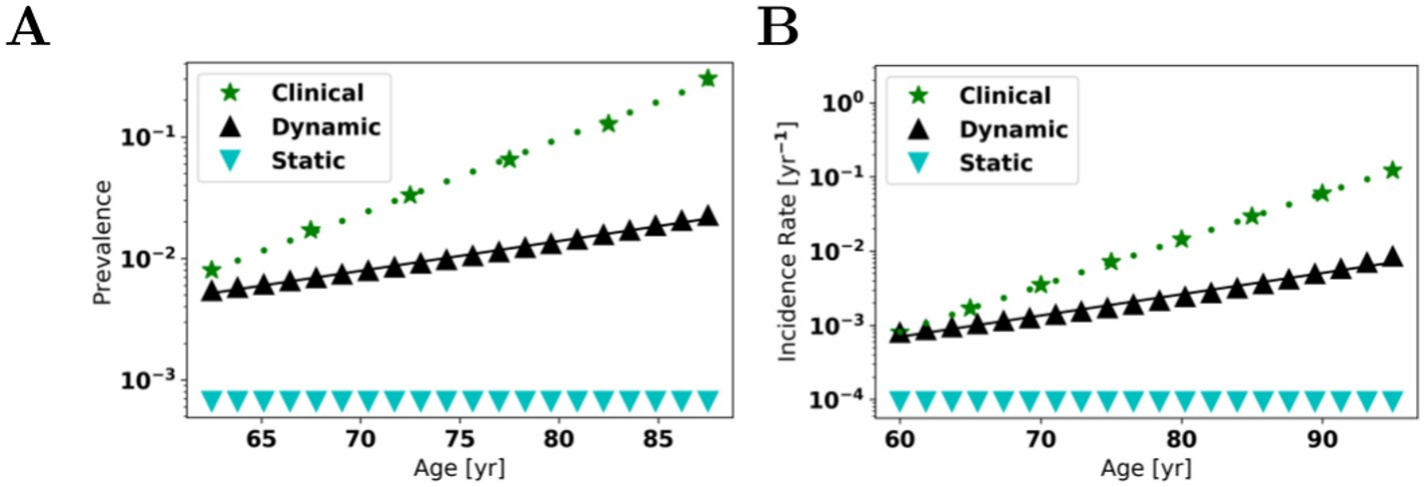
Incidence and prevalence. Comparison of static and dynamic models with clinical data for AD. The dotted green lines represent the line of best fit to clinical data [19, 34] on log-scale; The black solid lines are the lines of best fit to the dynamic model on log-scale. *A*: for prevalence, the clinical doubling time is 4.9 y and our dynamic model predicts 12 y. *B*: for incidence, the clinical doubling time is 4.9 y and our dynamic model predicts 11 y. The value *γ* is chosen so that clinical and dynamic model incidence agree at age 60.

Allowing the rates to vary, we can examine how the incidence and prevalence increase with age, which we depict in Fig 3. We choose *γ* so that the dynamic model incidence at age 60 matches clinical data, finding *γ* = 0.601. Using a linear best fit on the log-scale, our dynamic model predicts doubling times for prevalence and incidence of 12 y and 11 y, respectively. The fact that the model’s predictions are within a factor of 2−3 of the clinically observed times is encouraging. From (9)_2_ with *t*_1_ = 60 y and *t*_2_ = *T*_*L*_, our model predicts a lifetime risk of 2.4%.

### Gene dosage and Down’s Syndrome

#### Clinical data

Due to the under- or over-expression of particular genes, the production of monomers could be altered. For individuals with Down’s Syndrome, the trisomy of chromosome 21 results in life-long levels of APP that are ≈ 1.5 times that of normal individuals [14, 15] and an AD incidence at least 3 times higher [50]. In addition, Down’s Syndrome patients may present symptoms of dementia as early as age 40 [13].

Zigman et al. [51] have reported the cumulative incidence of AD within the general and Down’s Syndrome populations. From their work, we estimate that at age 70, the prevalence of AD is 4% in the general population and 65% in the Down’s Syndrome population. At age 80, the prevalences are 18% and 70%, respectively. From these data, we find that at ages 70 and 80, the ratios of the prevalences of AD among Down’s Syndrome individuals to the prevalence of AD among the general population are 16.25 and 3.89, respectively.

#### Model

Based on the ODE model, we can predict the ratios of prevalences between the Down’s Syndrome and general populations at ages 70 and 80. For the static model, these ratios are 2.24 and 2.24, respectively (the prevalence of the static model does not vary with ages above *T*_*D*_). For the dynamic model, the ratios are 3.07 and 3.15, respectively.

### Hippocampal volume (HV)

#### Clinical data

Changes in HV can take place during the aging process but these changes are particularly extensive in AD patients. Frankó et al. [52] used MRI to estimate HV in longitudinal studies of AD patients, mean age 75, patients with mild cognitive impairment (MCI), mean age 75, and controls, mean age 76. They found that HV decreased by averages of 42 mm^3^, 30 mm^3^, and 15 mm^3^ per year in the AD, MCI, and control groups, respectively. The average HVs reported at initial scans were 3934 mm^3^, 4127 mm^3^, and 4464 mm^3^, respectively. Using these data, we can estimate that around age 75, the three groups have approximate annual decreases in HV of 1.06% (AD), 0.727% (MCI), and 0.336% (control). We note some studies in cognitively normal individuals have failed to find significant differences in HV but did find statistically significant differences in some brain measures like the thicknesses of the entorhinal cortex and parahippocampal gyrus when subjects were classified into two groups A*β*^+^ and A*β*^−^ with a Pittsburgh Compound B (PiB) MRI scan [53].

In a study by Gordon et al. [54], participants received MRI and PiB/PET scans along with assays of tau and phosphorylated tau. Patients then were classified into four disease states reflecting the presence/absence of amyloid (A*β*^+^/A*β*^−^) and the presence/absence of CSF tau/phospho-tau, which were used as a proxy for neurodegeneration (ND^+^/ND^−^). We focus here on the states 0 (A*β*^−^/ND^−^) and 2 (A*β*^+^/ND^+^), which we consider normal or “AD.” The study found those in state 0 (mean age 63.4) had measured HVs of 7755 mm^3^ and those in state 2 (mean age 71.6) had measured HVs of 7063 mm^3^. Thus, the AD patients of mean age 71.6 had HVs that were only 91.1% as large as those without AD and mean age 63.4.

#### Model

In the ODE model, if we assume HV is proportional to *V*(*t*), the model yields estimates for HV changes over time. We test our static and dynamic models against the data described above. We find the dynamic model adequately describes cognitively normal individuals. To describe the HV rate of change in AD patients and the HV ratios at different ages, we need to scale *U*(*t*) (11)_1_ up by a factor *F* > 1. This then leads us to examine how a distribution of rate parameters within the population could influence clinical outcomes.

#### Static model (*κ*, *S* constant)

With static values, each year, the hippocampal volume should decrease by a rate $\frac{-V^{\prime }\left(t\right)}{V(t)}=\bar{U}=0.0154\%{\text{y}}^{-1}.$ This is a factor of ≈ 22 smaller than the typical loss of HV in non-AD patients. However, we would not expect the agreement to be strong because the static model does not include effects of aging upon rate constants like *S* and *κ*.

#### Dynamic model (*κ*, *S* time-dependent)

**Fig 4:**
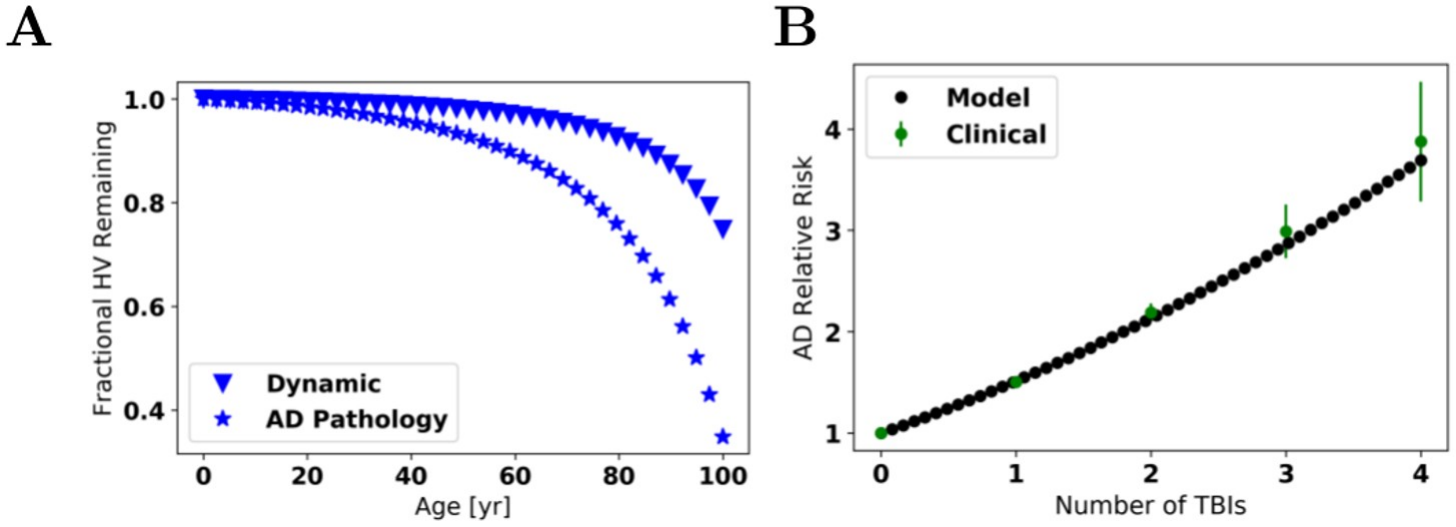
*A*: **Time dependence of HV** for the dynamic model with or without additional AD pathology. An HV of 1 is maximal. At age 75, the annual changes in hippocampal volume are −0.015% (static model, not shown), −0.29% (dynamic model), and −1.1% (AD pathology model—rates have been scaled to match this value). The HV ratio between those at age 71.6 (AD pathology) to age 63.4 (CN) is 0.859. We can also compare *within models*. The hippocampal volume ratios between age 71.6 to age 63.4 years are as follows: 0.999 (static), 0.984 (dynamic), and 0.944 (AD pathology). *B*: **Traumatic Brain Injury**. Our fit to clinical data [56] for the relative hazard rate $\hat{R}$ vs number of TBIs, *n*. The error bars represent one standard error. Model fit: $\hat{R}\left(n\right)={\left(1+an\right)}^{2}$ with $a=A/\bar{S}$ to be estimated. The fitted value is *a* = 0.231.

We compare the model predictions with clinical findings in Fig 4. We plot what the HV would look like under the dynamic model with the relative change (*V*′(*t*)/*V*(*t*) = −0.29%/y) at age *t* = 75 y. This agrees well (within ≈ 16%) with the rate of change of −0.336%/y in the control group of Frankó.

Our model attempts to describe the average patient and their resulting HV (or neuronal viability) over a lifetime. AD development is seen as a probabilistic event where the probability of developing AD depends on the amount of HV lost. Our dynamic model does not match the observed rate of −1.06%/y for the HVs in the Frankó AD patients (it is off by a factor of ≈ 3.65). One possible means of reconciling this is by assuming those with AD on average have *U*(*t*) values that are larger than the general population by a factor *F* = 3.65 so as to match −1.06%/y. This **AD-pathology model** with rescaled rate constants is also shown in Fig 4.

Given the dynamic model and the AD pathology model, we can compute the ratio in HVs between those at age 71.6 years (state 2^+^) to those of 63.4 years (controls). Taking the HV ratio of our AD pathology model at age 71.6 to the dynamic model at age 63.4 yields 0.859, which is close to the observed 0.911 ratio (with ≈ 6%).

#### Distributions of damage rates

We now ask whether the model allows for those with AD to have higher values of *U*(*t*) than the rest of the population. For simplicity, we study the static model and assume *U*(*t*) = *U*_0_, a constant, where *U*_0_ ∼ *g*(*u*) has a probability density function with mean value U* and standard deviation Σ*.

On one hand, it may seem obvious that if someone has AD, more neurons have been destroyed and an appreciably above average *U*_0_ is expected (*U*_0_ describes the rate of neuronal death). But even people with lower values of *U*_0_ can develop AD and, depending on the distribution, there could be many more people with average or below-average *U*_0_’s than those with *U*_0_’s that are above the average. Thus, it is not immediately obvious that AD patients will have above-average *U*_0_ values. This above-average *U*_0_ in the AD group turns out to be true, however, which we show in the S1 Text (Uncertainty Quantification and Damage Distributions section). It is in fact true even in the dynamic model when *U*(*t*) is *U*_0_ times an age-dependent scaling. In particular, within the AD and non-AD populations, the average *U*_0_ values are

$$\begin{array}{c} E\left[U_{0}|{\text{AD}}^{+}\right]\approx U^{*}+\frac{{\Sigma^{*}}^{2}}{U^{*}},\;E\left[U_{0}|{\text{AD}}^{-}\right]\approx U^{*}. \end{array}$$ (18)

Thus, those without AD on average have a “normal” *U*_0_ but those with AD on average have an above average *U*_0_. This would allow for consistency between our model and the requirement to scale *U*(*t*) to match AD-specific data.

Under these assumptions, we can be very specific with (18) about how spread out *U*_0_-values are within the population. We find that $\frac{{\Sigma^{*}}^{2}}{{U^{*}}^{2}}=F-1$ is most consistent with the data. Numerically, with *F* = 3.65, we find that the standard deviation to mean ratio is

$$\begin{array}{c} \Sigma^{*}/U^{*}\approx F^{*}=1.63. \end{array}$$ (19)

### Blunt force trauma

#### Clinical data

Whether the risk of developing AD definitively increases as a result of Traumatic Brain Injury (TBI) is not clear [55] as there are many factors at work: the nature of the trauma, its location, whether consciousness was lost, and whether TBI incidents are reported/remembered, etc. However, a more recent study by Fann et al. [56] does provide data for estimated hazard ratios of developing AD given a patient’s history of TBI and years since their first TBI. For our study, we focus upon the long-term risk of AD given the number of TBIs using their “model 1”, which adjusts for age, sex, marital status, and calendar period, but does not adjust for other comorbidities since the comorbidities may reflect physiological differences between individuals, which would require further modeling.

After an acute TBI, it has been noted that APP processing increases, resulting in increased A*β* production and deposition [57]. In studies on pigs with a head rotational acceleration injury, axonal damage, resulting in an accumulation of APP, has been noted 6 months after injury [58]. In humans, axonal damage and intra-axonal A*β* accumulation can last for years [58]. It should be noted that neprilysin, an A*β* degrading enzyme, also appears to be upregulated following TBI, which could counteract increased A*β* production. Olsson et al. [59] found that after TBI, the concentration of ventricular cerebrospinal fluid-A*β*(1–42) increased over the days following the event by ≈1073%. Likewise, in the days following the TBI, ventricular cerebrospinal fluid-*α*-sAPP increased by 1933%.

#### Model

For simplicity, for each TBI, we assume the rate *S* increases by a fixed amount *A*, without mitigating effects and *we work with the static model to avoid needing the age of a patient at each of their TBIs*. In the Discussion we comment on extending this to the dynamic model.

Assuming that for each TBI, the monomer production rate rises by a constant value, the relative hazard rate (relative to having no TBIs) after having *n* TBIs should be $\hat{R}={\left(1+n\frac{A}{\bar{S}}\right)}^{2}$ based on Eqs (13)_2_ and (15)_2_. This model has one free parameter, $a=A/\bar{S}$. After fitting, see Fig 4, we venture the idea that, very approximately, each TBI results in a long-term increase in the monomer production rate of approximately $0.231\bar{S}$. While the Olsson et al. [59] did not monitor A*β* concentration over years, given the massive (> 10-fold) increases in A*β* observed, a lifetime increase of 23.1% is not unrealistic. Were A*β* concentration to increase by a factor of 10, then, over that time window with such high A*β*-levels, the relative hazard rate would be 100!

### Spatial spreading of AD

**Fig 5:**
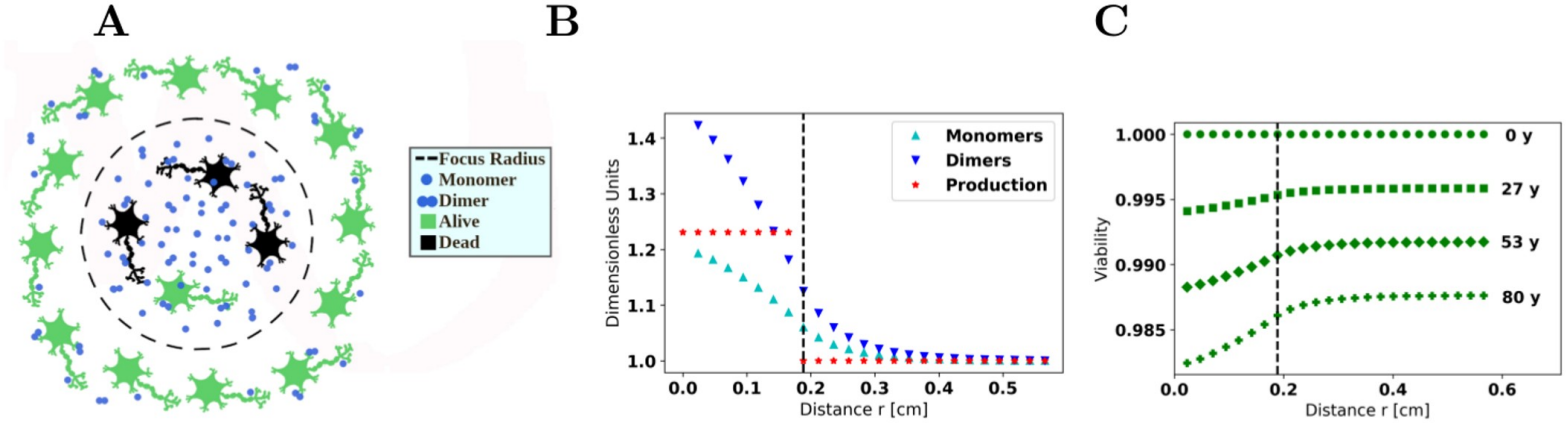
Spatial model. *A*: the excess monomer production is taken to be spherically symmetric. The distance (*x*−axis) denotes the distance from the center of the source. The dashed circle/lines represent the boundary where excess monomer production ceases. *B*: monomer and dimer concentrations, and monomer production rate, versus distance from center. These values have been nondimensionalized by $\bar{M}$, $\bar{D}$, and $\bar{S}$, respectively. *C*: viability at various ages plotted against position.

Using the PDE model, we gain insight into the effects of localized excesses of monomers. In an idealized, spherically symmetric geometry with constant rate constants, we consider a hypothetical scenario. We imagine that over a sphere radius of $2\bar{x}$, the monomer production rate is increased by an amount $0.231\bar{S}$, the characteristic increase that we speculate results from a TBI, and that this increased production remains constant over a lifetime. This results in a modest excess of monomers and dimers above their baseline values. In turn, this affects the viability of cells in that vicinity so that over a lifetime, cell damage is more pronounced nearer to the excess monomer production. We display the results in Fig 5. These results suggest that if a part of the brain is damaged, resulting in a local excess of monomers, the closer that region of damage is to neurons that are particularly important for memory, the more likely lifetime risk may be permanently elevated, even if these neurons were not originally damaged.

Another interesting result is the spatial extent over which monomer concentration is elevated. In the example, monomer production is increased by ≈ 20% over a radius of ≈ 0.2 cm. At the center, the monomer and dimer concentrations have risen by ≈ 20% and ≈ 40% over their baseline values. However, on the surface of the sphere, the monomer and dimer concentrations have already diminished to almost their baseline values.

### Uncertainty quantification

Here, we consider variability within the model including (1) how variations in the rate constants affect disease outcomes, i.e., *sensitivity*; (2), how a hypothetical *distribution of damage rates* within a population alter observable outcomes; and (3), how disease outcomes may be observed in finite population sizes.

To keep the analysis simpler, we will assume

$$\begin{array}{cc} U(t) & =\frac{\sigma \left(t\right)S^{2}\left(t\right)\nu \left(t\right)}{\kappa^{2}\left(t\right)\mu \left(t\right)}=U_{0}\Xi \left(t\right) \end{array}$$ (20)

$$\begin{array}{cc} \omega (t) & =\frac{\gamma \sigma \left(t\right)S^{2}\left(t\right)\nu \left(t\right)}{\kappa^{2}\left(t\right)\mu \left(t\right)}=\omega_{0}\Xi \left(t\right) \end{array}$$ (21)

where

$$\begin{array}{cccc} U_{0} & =\frac{\sigma \left(0\right)S^{2}\left(0\right)\nu \left(0\right)}{\kappa^{2}\left(0\right)\mu \left(0\right)}, & & \omega_{0}=\frac{\gamma \sigma \left(0\right)S^{2}\left(0\right)\nu \left(0\right)}{\kappa^{2}\left(0\right)\mu \left(0\right)} \end{array}$$ (22)

are the neuronal damage rate and the AD development rates at birth and Ξ(*t*) is a scaling factor. For the static model, Ξ(*t*) = 1. In the dynamic model, Ξ(*t*) increases with age.

From these assumptions and Eqs (8)_2_ and (9)_1_,

$$\begin{array}{c} P\left(t\right)=1-\text{exp}\left(-\omega_{0}\int_{\text{max}\{0,t-T_{D}\}}^{t}\Xi \left(s\right)\text{d}s\right),\;I\left(t\right)=\omega_{0}\Xi \left(t\right) \end{array}$$ (23)

are the full time-dependent solutions. With *S* and *κ* varying as prescribed in Eq (5)_1,2_, $\int \Xi \left(t\right)\text{d}t=\lambda_{\kappa }^{3}\left(\frac{t}{\lambda_{S}^{2}\lambda_{\kappa }}+\frac{{\left(\frac{1}{\lambda_{S}}+\frac{1}{\lambda_{\kappa }}\right)}^{2}}{1-t/\lambda_{\kappa }}+\frac{2(\frac{1}{\lambda_{S}}+\frac{1}{\lambda_{\kappa }})}{\lambda_{S}}\text{log}\left(1-t/\lambda_{\kappa }\right)\right)+C.$

Let *f* be a response variable that depends upon the quantity *q*. The *sensitivity* of *f* with respect to *q* shall be denoted by

$$\begin{array}{c} \Delta_{q}^{f}≔\frac{q}{f}\frac{\partial f}{\partial q}, \end{array}$$ (24)

and it is the ratio of the relative change in *f* to the relative change in *q*. Roughly it is the percentage that *f* changes when *q* increases by 1%.

#### Model sensitivity

There are various approaches to assess how the model behaves in the presence of perturbations to the parameters. We consider first *small* perturbations and describe how outcomes vary with a 1% change in a parameter value, for instance. Then we consider the possibility the AD development rate *ω* could vary substantially from our estimate. Owing to the large uncertainty in rate constants, this is possible: literature suggests wide ranges of values for $\bar{\mu }$ [42, 43]. However, as described in the S1 Text (Uncertainty Quantification and Damage Distributions section), the estimates we have are still valid in many of these cases.

#### Sensitivity analysis

Denoting *P*_65_ and *I*_65_ as the AD prevalence and incidence at age 65, for example, we find that $\Delta_{\omega_{0}}^{P_{65}}=0.997$, $\Delta_{\omega_{0}}^{I_{65}}=1$, $\Delta_{\gamma }^{\omega }=1$, $\Delta_{\bar{S}}^{\omega_{0}}=2$, $\Delta_{\bar{\kappa }}^{\omega_{0}}=-2$, $\Delta_{\bar{\mu }}^{\omega_{0}}=-1$, $\Delta_{\bar{\nu }}^{\omega_{0}}=1$, and $\Delta_{\bar{\sigma }}^{\omega_{0}}=1$.

#### Scaling AD development rate *ω*

**Fig 6:**
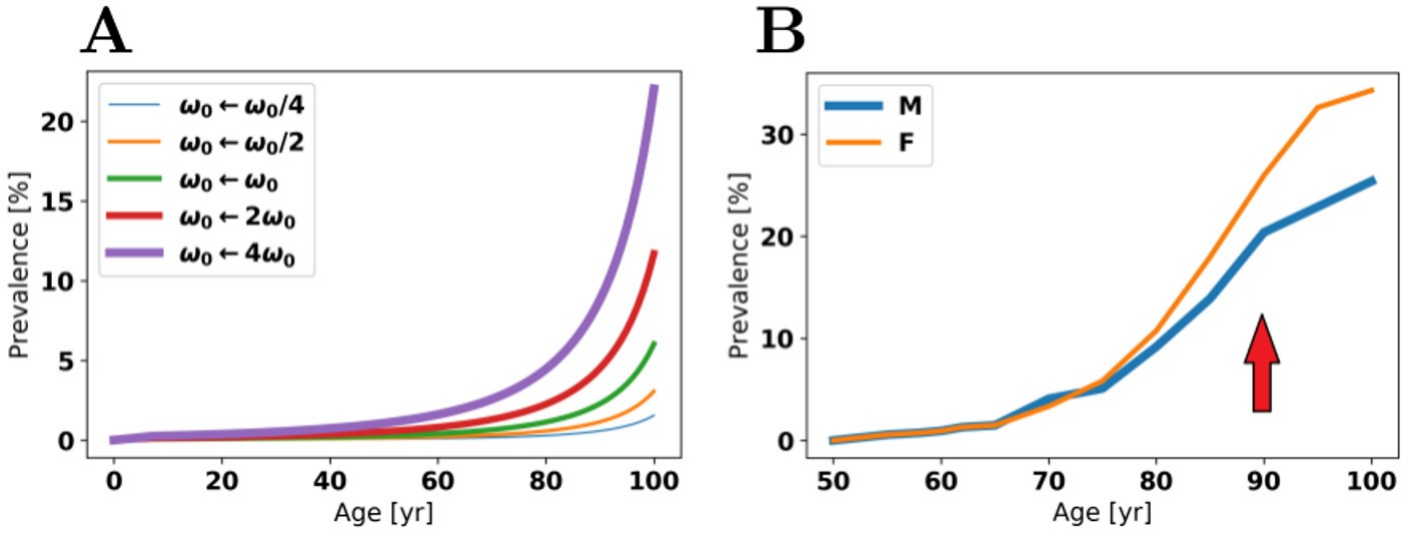
Significant variations of rate constants. *A*: the prevalence for the time-dependent model as *ω*_0_ is scaled. *B*: clinically observed prevalence of AD in males (M) and females (F) [60] with inflection point marked by arrow.

In Fig 6, we plot the prevalence for the time-dependent model by allowing *ω*_0_ to be scaled by several powers of 2. Roughly speaking, we find that the incidence goes up by a factor of 2 every time *ω*_0_ does. What is interesting is that if we consider clinical data for AD prevalence in males and females [60], the curves are different, possibly reflecting differences in *ω*_0_ between the two sexes. This data is also presented in Fig 6. The dynamic model, in contrast, does not saturate at a prevalence below 100%.

In clinical AD data [60], the rate of increase of prevalence appears to slow after around age 90, where we observe an inflection point (see arrow in Fig 6). It may be that relevant rate constants such as *S* and *κ* change differently much later in life than in our model. Thus, while the model may be mathematically valid up to λ_*κ*_ = 114 y, its qualitative description of prevalence is most descriptive up to age ≈ 90 y.

#### Distribution of initial damage rates *U*_0_

We consider an example system to illustrate the effects of distributions of *U*_0_ within the population. From Eq (19), we speculated there could be a probability distribution for *U*_0_ within the population with a mean of $\bar{U}$ and a specified standard deviation to mean ratio. We consider an example distribution given in Table 2. We note there are many more people who have below average damage rates than those with above average damage rates.

**Table 2:** Example distribution. The probability distribution here has that the mean *U*_0_ value is $\bar{U}$ and the standard-deviation to mean ratio is consistent with (19).

Value of *U*_0_	Probability
$\bar{U}/10$	0.765
$3.93\bar{U}$	0.235

We explore the effects of distributions of *U*_0_ values upon prevalence and incidence both analytically and through running stochastic simulations. We simulate a population of size 40, 000 over 90 years. These simulations are not deterministic. Results appear in the S1 Text (Uncertainty Quantification and Damage Distributions section). We find that prevalence and incidence are lower given a distribution of *U*_0_-values with a given mean $\bar{U}$ than when $\bar{U}$ is the only value for *U*_0_. We show mathematically [61] in the S1 Text that this effect (that when *U*_0_ is distributed about a mean, incidence and prevalence will be lower than or equal to the incidence and prevalence when *U*_0_ can only take one single value) is independent of the specific distribution. This is rather surprising as allowing a distribution of *U*_0_-values means that *U*_0_ can be larger than $\bar{U}$ in some individuals. But in the end, despite some having this larger rate, the prevalence and incidence end up being smaller. Put another way: if within a population there are people with a distribution of *U*_0_-values then if everyone in the population had a damage rate equal to the mean (and this would include lowering the damage rate of those with above-average values), prevalence and incidence would both increase! However, the difference between fixing $U_{0}=\bar{U}$ and having a distribution is small.

#### Effects of population size

From the stochastic simulations described in the preceding section, for a fixed initial population size *N*_0_, we can compute confidence intervals for observables like prevalence and incidence. We find the upper and lower bounds of the confidence intervals are very close to the mean value. As *N*_0_ → ∞, i.e., for large enough populations that have been observed, which is on the order of several million for AD, all simulations tend to yield the mean value, which we have already calculated (see S1 Text (Uncertainty Quantification and Damage Distributions section)).

## Discussion

### Quantitative predictions

We have developed a mathematical model for AD risk and progression based on the kinetics of A*β* oligomerization and clearance and its relationship to neuronal viability. We sought to combine data concerning oligomer kinetics and neuronal properties such as sensitivity to oligomers and microscale in the brain and infer their effect upon changes in brain physiology and AD development on a macroscale over a lifetime. Besides one free parameter, *γ*, the macroscale information was not used for fitting. As a result, agreement with clinical data is significant. Even qualitative agreement is significant. Even if precise values of the parameters are not known at the time, qualitative agreement could suggest the correct mechanisms are incorporated in the model. We summarize the results in Table 3 and discuss the comparisons below.

**Table 3:** Summary of model results. Each result is presented in this manuscript. Three significant figures are used as the model results come from formulas and the parameters were stored with 3 significant figures.

Phenomena	Clinical Data	Model Result
**HV rate of change**	−0.336%/y control age 76 [52]	*Predict* −0.29%/y
**HV rate of change**	−1.06%/y AD age 75 [52]	*Infer* AD group has higher damage rate by factor *F* = 3.63 and possible damage rate distribution
**HV ratios**	Ratio is 0.911 for AD group at age 71.6 to control at age 63.4 [54]	*Predict*: 0.859
**TBI**	Relative Incidence as function of number of TBIs [56]	*Assuming* each TBI increases monomer production by $0.231\bar{S}$, *nearly perfect match*
**Prevalence & Incidence**	Both double every 4.9 y [19, 34]	*Predict* doubling times of 12 y and 11 y, respectively
**Prevalence & Incidence**	Observed variation with age [19, 34]	Does not agree
**Lifetime risk**	17% from age 60 [49]	*Predict*: 2.4%
**Down Syndrome relative prevalence**	≈ 3 − 16 times higher [50, 51]	*Predict*: 3.07 − 3.15 times higher risk, depending on age
**Down Syndrome prevalence and relative prevalence**	Variation with age [51]	Does not agree
**Localized Increase of Monomer Production**	N/A	Quantitative description of monomer, dimer, and viability vs time near a focus of ≈ 1 mm^3^ increased monomer production

#### Hippocampal volume

The dynamic model predicts an annual rate of HV loss in cognitively normal individuals at age 75 to within 16% of the rate found in patients, thus successfully modeling the normal aging process and changes to HV over time [52]. This is very promising as only rate constants based on oligomer kinetics (monomer production rate, dimerization rate, etc.) and cell viability assays were used, yet the model accurately described large scale physiological changes taking place over years or decades. Our model then showed that the average neuronal damage rate could be higher on average among patients with AD. By assuming the damage rate is *F* = 3.63 times higher in AD patients, we were then able to describe the rate of HV loss per year in AD patients at age 75. We found that the ratio of brain volumes among those with mean age 71.6 y who had AD to those without AD with mean age 63.4 y differed from published values [54] by only 6%.

#### Traumatic brain injury

By assuming that each TBI increases monomer production by a fixed amount $0.231\bar{S}$ due to the quadratic dependence of incidence upon monomer concentration, our static model predicts that the relative incidence $\hat{R}$ with *n* TBIs is given by $\hat{R}\left(n\right)={\left(1+0.231n\right)}^{2}$. The agreement with clinical data is nearly perfect [56].

Similar results could be obtained with the dynamic model, but we would have to assume that $S\left(t\right)=\bar{S}\left(1+0.231n\right)\Xi \left(t\right)$. We could interpret this as *roughly* saying: monomer production scales with Ξ(*t*) due to increased *γ*-secretase activity over a lifetime and it is proportional to the amount of APP, which grows by 0.231 of its baseline value for each TBI. We note that TBI leading to AD could be mediated by proteins other than A*β*. As we remark in the Cell Viability, Incidence, and Prevalence section, although beyond the scope of our current work, the model could be modified to include such effects.

#### Prevalence and incidence

The dynamic model predicts AD prevalence and incidence double every 12 and 11 y, respectively, which is close (within a factor of ≈ 2 − 3) to the 4.9 y observed clinically for both [19, 34]. The factor of 2 − 3 is not concerning as the empirical formulas to model *S*(*t*) and *κ*(*t*) were as simple as possible (linear). The fact that over the age ranges clinically studied our model yields approximate exponential growth and that this growth rate is even within an order of magnitude of the clinical data shows promise.

With all of the constants from the oligomerization rate constants and neuronal sensitivity fixed, we only had one free parameter, *γ*, which was used to match incidence data at age 60. The doubling times for incidence (and prevalence, at least approximately), are *independent of γ*. Thus, the comparison here is entirely based on the extrapolation of cell-scale phenomena to the population scale studied over decades. The model and clinical incidence and prevalence do not agree because the doubling times do not match and thus the errors will get worse with age. The agreement between model and clinical incidence at age 60 is due to the choice of *γ*.

#### Lifetime risk

The lifetime risk of someone 60 y of age developing AD has been reported to be 17% [49]. Our dynamic model predicts a value of 2.4%. The prediction is only accurate to within an order of magnitude. The main reason for the magnitude of this difference is mathematical: if the incidence values are not accurate in the model, the accumulated risks we calculate will not be valid either.

#### Down’s syndrome

Those with Down’s Syndrome, who express ≈ 50% more APP than normal individuals, will have an AD prevalence that is much more than 50% higher. This is a quantitative mystery. The relative prevalence ranges from 3 to perhaps 16 [50, 51]. Our model suggests that those with Down’s Syndrome have a relative prevalence of 3.15 at age 80. The fact that our model accurately describes the ≳ 3-folder greater prevalence is an accomplishment. In general, we find that if monomer production increases by a factor Ω^2^ then incidence and prevalence increase by at least ≈ Ω^2^ (see S1 Text (Uncertainty Quantification and Damage Distributions section)). This is a lower bound that ignores age-dependent changes in kinetic rate constants. By including this quadratic dependence with a more rapid increase of *γ*−secretase activity observed in Down’s Syndrome patients, we have gained insights into the much higher prevalence.

#### Spatial spreading of AD

We were able to consider a hypothetical scenario where monomer production was increased over a millimeter scale for a lifetime. Our model provided quantitative insights into the monomer and dimer concentrations and the cell viability as a function of distance from the excess production at various ages. It will be interesting to see if this prediction from the model is observed in patients. To our knowledge, such information is not yet available.

#### Age-dependent toxicity *σ*

As a further exercise, we consider how the model agreement can improve with a linear, age-dependent toxicity function *σ*(*t*). Our model applies to the general possibility that rate constants other than *S* and *κ* vary slowly with age. We take *σ*(*t*) = *σ*_0_(1 + *t*/λ_*σ*_) with *σ*_0_ a constant. We thus assume *σ* increases with age. Data do suggest that older neurons are more sensitive to A*β* oligomers [62]. Studies have also found that with age, energy production and DNA repair may be reduced in cells [63]; and within the brain, neurons experience increased oxidative stress, perturbed energy homeostasis, and accumulations of damaged proteins [64]. Indeed, *σ* increasing with age is likely.

What we find is that if λ_*σ*_ = 9.01 y, $\sigma_{0}=0.107\bar{\sigma }$, and *γ* were set to 0.732 from its present 0.601, then we recover the HV data as before, AD prevalence doubles every 8.9 y, and AD incidence doubles every 10 y over the range of ages we fit previously. This agrees even better with the clinical doubling times of 4.9 y for both incidence and prevalence. Details are presented in the S1 Text (Uncertainty Quantification and Damage Distributions section).

### Implications

If the model’s rate constants accurately represent those occurring in vivo then, by Eqs (11)_2_ or (15)_2_, the model predicts that the rate of neuronal death (and AD incidence) is proportional to *S*^2^ (*S* is the A*β* monomer production rate), *ν* (dimerization rate), and *σ* (cell sensitivity to dimers) and inversely proportional to both *κ*^2^ (*κ* is the A*β* monomer clearance rate) and *μ* (dimer dissociation rate). The quadratic dependencies are most significant: If *S* increases by a factor of 2 (or if *κ* decreases by a factor of 2), the neuronal death rate quadruples. While it is logical that lower monomer production and increased clearance would be therapeutically beneficial, our study here gives a *quantitative* (quadratic) basis for the effects of these treatment objectives. The other factors *σ*, *ν*, and *μ* only influence the death rate in direct proportion (or inverse proportion) to their value. For instance, if *μ* doubles, the death rate goes down by a factor of 2.

The ODE model accounts for the loss of HV over a lifetime and the changes in AD risk associated with APP gene dosage. If APP gene expression changes by a factor Ω then relative risk scales by *at least* Ω^2^. The static ODE model, through fitting for a free parameter, allows us to precisely describe how TBI increases one’s lifetime risk of AD if each TBI increases the monomer production rate by $0.231\bar{S}$. The PDE model provides an understanding of how A*β* concentrations and cell viability could vary over millimeter scales, leading us to speculate that the location of a TBI may influence the increased lifetime risk of AD: even if a brain injury occurs away from a neuron that is especially important for memory, our model suggests that the closer the injury is to such a neuron, the greater the long-term AD risk. Efforts to reduce excess A*β* production near a site of injury could reduce AD risk.

Through uncertainty quantification, we found that variations in the neuronal damage rate and AD development rates within people can give rise to surprising phenomena. For example, if the mean value of the damage rate is fixed within the population then populations with *distributions* of damage rates (by which we mean not all the damage rates are the same) will have lower prevalence and incidence than populations where everyone’s damage rate is precisely the *mean* value. In addition, the mean damage rate among the AD population, even if most of the people in the population have damage rates below the mean, will be higher than the mean. Through a comparison between male and female AD prevalence data, it is that possible some differences in prevalence stem from males and females having different AD development rates *ω*.

### Model improvements

We have shown that a model comprising of only a few mechanisms and assumptions is able to recapitulate many observed features of AD and aging. However, as would be expected for a new model, there are areas where the model is not accurate, such as the values of incidence and prevalence of AD year-by-year. Some natural next steps to improve the model could include accounting for: (1) the myriad of enzymes involved in A*β* metabolism; (2) genetic factors; (3) variations between brains; (4) cell repair; (5) coupling monomer production rates with the health/viability of the cells; (6) distinguishing the unique contributions of A*β*40 and A*β*42 to the pathogenesis of AD (both were considered equivalent); (7) expanding anatomical considerations from just interstitial fluid to the brain parenchyma and its distinct regions, ideally accounting for stereotypical spreading of disease (Braak staging); (8) accounting for differences in A*β*-induced toxicity among different neuronal cell types and brain regions; and (9) including contributions of glial cells and microglia.

We also would like to include more data to better account for nonlinearities in model results. As a heuristic example, we found that with static rate constants, the HV of a subject of age *t* would scale with ${\text{e}}^{-\bar{U}t}$. Most studies had a distribution of subject ages and HVs. For our models, we used the mean age for *t* as the time variable and the mean HV as a target volume as we did not have access to each subject’s data. However, *it is not generally true that the mean value of a nonlinear function evaluated at a series of inputs is equal to the nonlinear function evaluated at the mean value of the inputs*.

Most of our results come from the ODE model, which describes the brain as a homogeneous volume or describes the case in which the diffusion of monomers and dimers is infinite. The PDE model offers more opportunity to explore spatial effects. While our present PDE analysis did not consider the finiteness of the brain, the model could be adapted to describe boundary conditions, such as the blood brain barrier, and differences in brain compartments, such as location-dependent rate constants, varying diffusivities, etc.

In many cases, the model’s limitations stem from a lack of data pertaining to in vivo measurements of A*β* kinetics and oligomer toxicity. It would be of particular interest if the modeling assumptions and assumed rate constants could be validated clinically. However, one of the benefits of such a simple model is that many results can be obtained and understood from simple formulae, which could easily become intractable, and inaccurate (overfitted), with more elements and systems included. We expect many of the results presented would still hold, even if the rate constants changed significantly—provided the same processes can be deemed negligible from the larger-scale system behavior. In this sense, the model is quite robust even considering its limitations.

There are numerous other factors that have been implicated in AD risk and pathogenesis including diet, exercise, mood, brain activity, education, and sleep quality (impaired glymphatic clearance of A*β* monomers during sleep [65, 66] may reduce *κ*, which would accelerate neuronal damage). Of course, many other factors likely exist about which we are unaware. These also could influence the model’s rate parameters so that some individual’s exhibit a faster neuronal death rate and increased risk for AD at every age. We expect our model to be used to conceive of new experiments in clinical and basic science settings and to be modified as the results of these experiments become available—a ping-pong effect.

## Conclusions and future work

We have developed a simple mathematical model describing the time dependence of development of AD and the contributions of A*β* monomers, dimers, and trimers to it. The model produces explicit equations whose solutions are consistent with clinical features of disease development and allow for interpretation of individual terms and rate constants. For example, the ratio of monomer production to monomer clearance, *S*/*κ*, is a term that is highly significant, suggesting that its reduction would lessen disease risk and slow progression. The model serves as a starting point for numerical simulations and in silico studies. Most importantly, the fact that such a complicated disease process can be simplified so much and produce accurate, clinically verified predictions suggests that the model can be used to test existing, and yield new hypotheses about disease causation. This would be especially valuable for studying aspects of AD for which little experimental data are available or the application of experimental or clinical methods of study is impractical. For example, the model could be used to explore the effects of predetermined numbers and magnitudes of TBIs on localized increased expression of APP and A*β* and consequent disease initiation and progression.

## Supporting information

S1 TextThis file contains the parameter estimation and mathematical analysis for our study.**Table A**: Viability Data. Values were estimated from graphs published by Lambert *et al*. [30] and Cizas *et al*. [31]. Standard errors for the Lambert data in the control (oligomer concentration of 0) are based on a worst-case estimate. The figure markings obscured the error bars and we chose the half-width of the largest marker as the standard error as part of the calculation. The study of Lambert et al. did not specify whether the error bars displayed were standard errors or standard deviations. We assume standard errors. Such variations only change *σ* by a modest scaling factor. **Table B**: Dimensionless parameters. With *ϵ* ≪ 1 chosen, these serve as constants for the asymptotic calculations. For the values displayed, we use $\bar{S}={\bar{S}}^{G}$. All parameters except for *ϵ* are *O*(1). The bottom parameters ensure that the slow timescales over which *κ* and *S* change are on the scale of 1/*ϵ*^2^. **Fig A**: Viabilities at various oligomer concentrations after 24 hours. We fit the model to viability data [30, 31]. The errors bars represent two standard errors. **Fig B**:*U*_0_ values conditioned on disease. The mean value of *U*_0_ is plotted among patients with AD, *AD*^+^ and patients without AD, *AD*^−^ as a function of age. The approximations derived above are extremely accurate, even in the dynamic model. *A*: static model. *B*: dynamic model. **Fig C**: Confidence Windows. The solid line is the simulation mean and the dashed lines represent the boundaries of the 95% confidence window. *A/B*: with *U*_0_ fixed and static; *C/D*: with *U*_0_ fixed and dynamic; *E/F*: with *U*_0_ from example distribution and static; *G/H*: with *U*_0_ from example distribution and dynamic. **Fig D**: Stochastic Trajectories. A few random trajectories of prevalence and incidence for the entire population. *A/B*: with *U*_0_ fixed and static; *C/D*: with *U*_0_ fixed and dynamic; *E/F*: with *U*_0_ from the example distribution and static; *G/H*: from *U*_0_ from example distribution and dynamic. **Fig E**: Incidence Model. *A*: *U*_0_ fixed and static; *B*: *U*_0_ fixed and dynamic; *C*: *U*_0_ from example distribution and static; *D*: *U*_0_ from example distribution and dynamic. Prevalence Model. *E*: *U*_0_ fixed and static; *F*: *U*_0_ fixed and dynamic; *G*: *U*_0_ from example distribution and static; *H*: *U*_0_ from example distribution and dynamic. Incidence variation with *ω*_0_. *I*: incidence for dynamic model with *ω*_0_ varying. **Fig F**: Age-Dependent Toxicity. *A*: prevalence curve [19]. *B*: incidence curve [34]. *C*: HV curve.(PDF)
