# Supplementary material for: From reaction kinetics to dementia: A simple dimer model of Alzheimer’s disease etiology
Source: PLoS Comput Biol. 2021 Jul 19;17(7):e1009114. doi: 10.1371/journal.pcbi.1009114 (PMC8321409; doi:10.1371/journal.pcbi.1009114)
Supplement: S1 Text — Table A: Viability Data. Values were estimated from graphs published by Lambert et al. [30] and Cizas et al. [31]. Standard errors for the Lambert data in the control (oligomer concentration of 0) are based on a worst-case estimate. The figure markings obscured the error bars and we chose the half-width of the largest marker as the standard error as part of the calculation. The study of Lambert et al. did not specify whether the error bars displayed were standard errors or standard deviations. We assume standard errors. Such variations only change σ by a modest scaling factor. Table B: Dimensionless parameters. With ϵ ≪ 1 chosen, these serve as constants for the asymptotic calculations. For the values displayed, we use S¯=S¯G. All parameters except for ϵ are O(1). The bottom parameters ensure that the slow timescales over which κ and S change are on the scale of 1/ϵ2. Fig A: Viabilities at various oligomer concentrations after 24 hours. We fit the model to viability data [30, 31]. The errors bars represent two standard errors. Fig B:U0 values conditioned on disease. The mean value of U0 is plotted among patients with AD, AD+ and patients without AD, AD− as a function of age. The approximations derived above are extremely accurate, even in the dynamic model. A: static model. B: dynamic model. Fig C: Confidence Windows. The solid line is the simulation mean and the dashed lines represent the boundaries of the 95% confidence window. A/B: with U0 fixed and static; C/D: with U0 fixed and dynamic; E/F: with U0 from example distribution and static; G/H: with U0 from example distribution and dynamic. Fig D: Stochastic Trajectories. A few random trajectories of prevalence and incidence for the entire population. A/B: with U0 fixed and static; C/D: with U0 fixed and dynamic; E/F: with U0 from the example distribution and static; G/H: from U0 from example distribution and dynamic. Fig E: Incidence Model. A: U0 fixed and static; B: U0 fixed and dynamic; C: U0 from example distr [file pcbi.1009114.s001.pdf]

From reaction kinetics to dementia: a simple dimer model of Alzheimer's disease etiology

Michael R. Lindstrom[1]*, Manuel B. Chavez[1], Elijah A. Gross-Sable[1], Eric Y. Hayden[2], David B. Teplow[2,3]

1 Department of Mathematics, University of California, Los Angeles, California, United States
2 Department of Neurology, David Geffen School of Medicine at the University of California, Los Angeles, California, United States
3 Molecular Biology Institute and Brain Research Institute, University of California, Los Angeles, California, United States

* mikel@math.ucla.edu

Supporting Information

Table A: Viability Data. Values were estimated from graphs published by Lambert *et al.* [2] and Cizas *et al.* [3].Standard errors for the Lambert data in the control (oligomer concentration of 0) are based on a worst-case estimate. The figure markings obscured the error bars and we chose the half-width of the largest marker as the standard error as part of the calculation. The study of Lambert et al. did not specify whether the error bars displayed were standard errors or standard deviations. We assume standard errors. Such variations only change σ by a modest scaling factor.
Table B: Dimensionless parameters. With $\epsilon \ll 1$ chosen these serve as constants for the asymptotic calculations. For the values displayed, we use $\bar{S} = \bar{S}^G$. All parameters except for ϵ are $O(1)$. The bottom parameters ensure that the slow timescales over which κ and S change are on the scale of $1/\epsilon^2$.
Fig. A: Viabilities at various oligomer concentrations after 24 hours. We fit the model to viability data [2, 3]. The errors bars represent two standard errors.
Fig. B: U_0 values conditioned on disease. The mean value of U_0 is plotted among patients with AD, AD^+ and patients without AD, AD^- as a function of age. The approximations derived above are extremely accurate, even in the dynamic model. *A:* static model. *B:* dynamic model.
Fig. C: Confidence Windows. The solid line is the simulation mean and the dashed lines represent the boundaries of the 95% confidence window. *A/B:* with U_0 fixed and static; *C/D:* with U_0 fixed and dynamic; *E/F:* with U_0 from example distibution and static; *G/H:* with U_0 from example distribution and dynamic.
Fig. D: Stochastic Trajectories. A few random trajectories of prevalence and incidence for the entire population. *A/B:* with U_0 fixed and static; *C/D:* with U_0 fixed and dynamic; *E/F:* with U_0 from the example distribution and static; *G/H:* from U_0 from example distribution and dynamic.
Fig. E: Incidence Model. *A:* U_0 fixed and static; *B:* U_0 fixed and dynamic; *C*: U_0 from example distribution and static; *D:* U_0 from example distribution and dynamic. Prevalence Model. *E:* U_0 fixed and static; *F:* U_0 fixed and dynamic; *G*: U_0 from example distribution and static; *H:* U_0 from example distribution and dynamic. Incidence variation with ω_0. *I:* incidence for dynamic model with ω_0 varying.
Fig. F: Age-Dependent Toxicity. *A:* prevalence curve [21]. *B:* incidence curve [22]. *C:* HV curve.

Parameter Estimation

We present our analyses to estimate the model parameters. In general, we seek to estimate parameters that are representative of a healthy brain. We understand that no two brains, let alone no two people, are exactly alike, therefore defining the parameters for a "healthy brain" is formally impossible. Nevertheless, for the purpose of creating a model, we have made our best estimates based on what information *is* available in the literature. We would hope that these estimates are within an order of magnitude of "formally representative."

Viability Modeling

One important metric for brain health is cell viability, which we denote $V(t)$ and define to be the number of viable neurons per unit volume, *relative to the optimal value in a healthy brain with no neuronal death.* Through aging and various insults, the value of V will decrease. We let D represent the concentration of dimers. For a given neuron, we denote $T > 0$ to be its age when it dies. We use survival analysis and hazard functions to model this [1].

Over a short time window δt, we assume

$$\Pr(t < T \leq t + \delta t) | T > t) = \sigma D \delta t$$

for some $\sigma > 0$. In other words, given that a cell lives as long as time t, the probability it dies over the next interval of length δt is proportional to δt (and to the dimer concentration). Alternatively, we can model cell death as an instantaneous Poisson (memoryless) process with rate σD. As a differential equation, we have

$$\frac{\mathrm{d}V}{\mathrm{d}t} = -\sigma D V. \tag{1}$$

To estimate σ, we choose two experiments [2,3] from the literature studying oligomer toxicity on neurons. There are many papers that study this phenomena, but most study only cultured neurons in isolation or in the presence of unrealistically high Aβ concentrations. Lambert *et al.* measured cell death in mouse brain slice cultures treated with various levels of oligomers for 24 hours. Cizas *et al.* performed similar experiments but with mixed neuronal-glial cell cultures. For our fitting, we assume:

- The cell viability V begins at maximum $V = 1$.
- The experiments directly measure V (in expectation, the fraction of surviving cells should indeed be V).
- General oligomer (dimers, trimers, n-mers) toxicity and dimer toxicity are the same.
- The values σ and D do not vary over the duration of the experiment.
- Cell death is induced only by the dimers and thus, at time t, we have $V(t) = \exp(-\sigma D t)$.

After estimating values from their respective published graphs, converting cell death to cell survival in Lambert *et al.*, and normalizing both datasets by the survival of their respective controls (see Table A), we fit for σ in (1) with a Maximum Likelihood estimate [4] (see Fig A). We obtain $\bar{\sigma} = 4.9 \pm 0.4$ M^{-1} s^{-1} (SE) as the characteristic dimer toxicity.

Oligomer Concentration [M]	Survival Fraction	Standard Error	Source
0	1	0.0256	Lambert
5×10^{-9}	0.862	0.0684	Lambert
5×10^{-8}	0.822	0.0649	Lambert
5×10^{-7}	0.806	0.0572	Lambert
5×10^{-6}	0.729	0.0890	Lambert
0	1	0.0598	Cizas
2.5×10^{-7}	1	0.0762	Cizas
5×10^{-7}	0.849	0.0918	Cizas
1×10^{-6}	0.275	0.0975	Cizas
2×10^{-6}	0.120	0.106	Cizas

Table A. Viability Data. Values were estimated from graphs published by Lambert *et al.* [2] and Cizas *et al.* [3].Standard errors for the Lambert data in the control (oligomer concentration of 0) are based on a worst-case estimate. The figure markings obscured the error bars and we chose the half-width of the largest marker as the standard error as part of the calculation. The study of Lambert et al. did not specify whether the error bars displayed were standard errors or standard deviations. We assume standard errors. Such variations only change σ by a modest scaling factor.

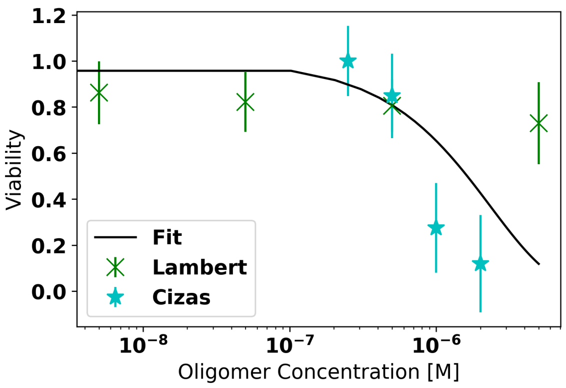

Fig A. Viabilities at various oligomer concentrations after 24 hours. We fit the model to viability data [2, 3]. The errors bars represent two standard errors.

To provide the survival fractions of Table A, we divided the survival rates from the experiments by their respective controls and added errors in quadrature to report the standard errors.

We note that we could allow V to be spatially dependent as well due to D potentially varying in space. Equation (1) would be unchanged, however, i.e., there would not be any explicit spatial dependence and all spatial variation would be implicit through the varying concentration of dimers.

It is important to remark that the effects of oligomer toxicity are significantly smaller in the Lambert study. Fitting individually, we find that with Lambert alone, the value is $\bar{\sigma} = 1.0 \pm 0.1$ M^{-1} s^{-1} (SE) and with Cizas alone, the value is $\bar{\sigma} = 8.9 \pm 0.6$ M^{-1} s^{-1} (SE). Changing σ up or down by factor of 2 would not alter our main results as we operate only with representative constants for the parameters and the formal asymptotic analysis done is robust, i.e., results do not appreciably change, with these sorts of perturbations. There is one notable exception: varying $\bar{\sigma}$ would have a similar effect to varying $\bar{D}$ in that the neuronal death rate would change and, in the HV study, the rate of change of volume would be different. However, the rest of the model has a dependence upon γ, which would then compensate for a changed $\bar{\sigma}$.

Incidence and Prevalence

For this modeling, we assume the brain tissue of interest (such as the hippocampus) has a uniform distribution of monomers and oligomers and a uniform viability measure $V(t)$. We begin by considering $H(t)$, the "survivorship function," being the "healthy" fraction of the population still alive at age t that does not have AD. We have

$$H(t) = \Pr(\text{AD has not developed by age } t | \text{alive at age } t).$$

We again use a survival analysis. Over a short time interval, δt, we assume

$$\Pr(\text{AD develops in } (t, t+\delta t] | \text{no AD up to time } t) = \gamma \frac{V(t) - V(t+\delta t)}{V(t)},$$

i.e., the probability someone develops AD in the interval $(t, t+\delta t]$ if they did not have it up to time t is proportional to the percentage of remaining neurons lost over the interval $(t, t+\delta t]$, where $\gamma > 0$ is a dimensionless proportionality constant that we expect to be on the order of unity. This is motivated by the fact that just a small number of neurons may be highly influential in memory formation [5] and we are describing the probability one of these influential neurons dies over the interval of width δt. In expectation, and with infinitesimal time steps,

$$\frac{\mathrm{d}H}{H} = \gamma \frac{\mathrm{d}V}{V}.$$

With $H(0) = 1$ and $V(0) = 1$, the solution is that

$$H(t) = V(t)^{\gamma}.$$

To model prevalence, we note that Alzheimer's patients live, on average, $T_D = 7.1$ years after diagnosis. Without specifically modeling the factors that lead to mortality, we assume that all Alzheimer's patients die T_D years following diagnosis. This factor is relevant in computing the prevalence, $P(t)$, which should be the fraction of the population (AD and non-AD) still alive at age t that has AD. This can be expressed as

$$P(t) = \frac{H(t - T_D) - H(t)}{H(t - T_D)}.$$

Neglecting the edge case of $t < T_D$, the numerator is the fraction of patients with AD within the population who are still alive at time t (they cannot be diagnosed earlier than $t - T_D$). The denominator is all people still alive, with and without Alzheimer's disease (to still be alive, they must develop AD later than $t - T_D$). The incidence (fractional rate people are diagnosed per unit time), $-H'(t)/H(t)$, is

$$I(t) = \gamma\sigma D.$$

A more rigorous derivation of the prevalence and incidence is provided later.

Monomer and Dimer Concentrations

We denote parameters of interest with a bar to indicate a characteristic value in a healthy brain.

Monomer Production Rate $\bar{S}$: The monomer production rate for Aβ42 is approximately 330 fM s^{-1} [6]. Combining this with the fact that a typical Aβ42/Aβ40 ratio is 0.1 [7], assuming this ratio also reflects their production rates, and focusing solely upon these polymers, we estimate the Aβ monomer production rate is 3.63×10^{-12} M s^{-1}.

Assuming that monomer production rate is proportional to APP concentration, with a 50% increase in APP concentration [8, 9], we find $\bar{S}^D = 5.45 \times 10^{-12}$ M s^{-1}.

It has been found that β-secretase activity increases with age [10]. One group reported an increase of $\approx 65\%$ over 100 years [11]. Fukumoto *et al.* [12] reported that in AD patients the increase was as much as 63% relative to normal controls. These authors also found that β-secretase activity increases by 58% over 50 years in Down's Syndrome patients but that α-secretase (the enzyme responsible for cleaving APP to release truncated peptides of Aβ) activity remains relatively constant throughout life for those with and without Down's Syndrome. We assume that monomer production rate is linearly proportional to β-secretase activity. This leads to values of $\lambda_S^G = 4.85 \times 10^9$ s and $\lambda_S^D = 2.72 \times 10^9$ s where, for example, we model monomer production in the general population as $\bar{S}^G(1 + t/\lambda_S^G)$.

Monomer Clearance Rate $\bar{\kappa}$: Aβ monomers are cleared at a rate $\kappa = 5.07 \times 10^{-5}$ s^{-1} for subjects of age 30 and 2.05×10^{-5} s^{-1} for subjects of age 80 [13]. We assume a linear decay in κ throughout life. This leads to $\lambda_\kappa = 3.61 \times 10^9$ s.

Dimer Concentration $\bar{D}$: From Lue et al., the range of total soluble Aβ was measured both for Aβ42 and Aβ40. The average concentrations of these peptides were 0 and 1.9 pg/g, respectively, for healthy people, and 15.5 and 66.5 pg/g, respectively, for those with AD [14]. Using molecular weights of 4514 g/mol for Aβ42 and 4329.9 g/mol for Aβ40, and assuming brain tissue has a density of approximately 1 g/cm^3, this results in dimer concentrations of 2.19×10^{-13} M for normal individuals and 9.16×10^{-12} M for AD patients. We choose $\bar{D} = 1$ pM, which is approximately the geometric mean of the two measurements for modeling.

Extra Assumptions. To estimate additional parameters, we make a series of assumptions:

1. monomers and dimers are in rapid equilibrium so in describing the dimer concentration, we always have that $\mu D \approx \nu M^2$; and
2. the loss of monomers due to forming higher-order structures (trimers and higher) is negligible in comparison to the loss due to clearance so that, in combination with the previous assumption, $\bar{M} = \bar{S}/\bar{\kappa}$.

Monomer Concentration $\bar{M}$: From assumptions 1 and 2, we deduce $\bar{M} = \bar{S}/\bar{\kappa} = 5.88 \times 10^{-8}$ M.

Ratio of Monomer-Monomer Combination to Dimer Dissociation Rates $\varrho = \bar{\nu}/\bar{\mu}$: By assumptions 1-2, we have that $\bar{\nu}/\bar{\mu} = 287$ M^{-1}.

Estimating $\bar{\mu}$ and $\bar{\nu}$: Experiments measuring $\bar{\mu}$ [15] have found $\bar{\mu} = 12700$ s^{-1}, but from experiments observing the conversion of oligomers to monomers, the time scale is on the order of days (so $\bar{\mu} \approx 10^{-5}$ s^{-1}) [16]. There is thus a large range of possible values for $\bar{\mu}$. We take the geometric mean of these two values to infer $\bar{\mu} = 0.4$ s^{-1}. Then from the ratio ϱ, we compute $\bar{\nu} = 115$ M^{-1} s^{-1}.

We note that while obtaining estimates for both $\bar{\nu}$ and $\bar{\mu}$ is an interesting exercise, in our model, the disease dynamics depend upon their ratio and not the values individually.

Diffusion Coefficients

Estimates for diffusivities of monomers and dimers are 1.4×10^{-6} cm^2/s and 1.1×10^{-6} cm^2/s, respectively [17]. The tortuosity of brain tissue is ≈ 1.6 [18]. The observed diffusivity of a chemical species $\mathcal{D}^*$ is related to its unimpeded diffusivity $\mathcal{D}$ and the tortuosity of its environment ι by

$$\mathcal{D}^* = \mathcal{D}/\iota^2.$$

This results in the diffusivities used in our study.

Mathematical Details

Here we provide some of the mathematical steps done to arrive at the results presented in the main body of the paper.

Negligible Higher Order Oligomers

The concentration of HOOs (trimers and above) is negligible within our parameter regime, thus they are not included in the model. This is a mathematical justification that supplements the focus upon dimers based on a quantitative measurements [19]. We shall denote O_j to be the concentration of oligomers of order j. Thus $M = O_1$ and $D = O_2$ as special cases. We assume that oligomer growth and loss is done through monomer addition/loss; furthermore, we assume that the oligomer growth rate (addition of a monomer) and oligomer dissociation rate (loss of a monomer) are given by those of the dimerization rate ν and dimer dissociation rate μ. Then in the absence of diffusion (as we are looking for represenative scales), we have

$$\begin{aligned}
\dot{O}_1 &= S - \kappa O_1 - 2\nu O_1^2 + 2\mu O_2 - \nu O_1 \sum_{j=3}^{\infty} O_j + \mu \sum_{j=3}^{\infty} O_j \\
\dot{O}_2 &= \nu O_1^2 - \mu O_2 - \nu O_1 O_2 + \mu O_3 \\
\dot{O}_j &= \nu O_1 (O_{j-1} - O_j) + \mu (O_{j+1} - O_j), \quad j = 3, ..., \infty
\end{aligned}$$

where the dot represents a time derivative. If we assume that $O_j \downarrow 0$ as $j \to \infty$, i.e., the concentration of oligomers of arbitrarily large size tends to 0 and that the system

can reach an equilibrium state, then at the equilibrium, by computing $\sum_{j=3}^{\infty} \dot{O}_j$, which is the rate of change of the total concentration of HOOs, we have

$$\nu O_2 O_1 - \mu O_3 = 0 \implies O_3 = \frac{\nu O_1}{\mu} O_2$$

and with this, in general,

$$O_j = (\frac{\nu O_1}{\mu})^{j-2} O_2, \quad j = 3, ..., \infty.$$

With $O_1 = \bar{M}$, we have that $O_3, O_4, ...$ are negligibly small. Asymptotically, $\frac{\nu O_1}{\mu}$ is on the order of ϵ^2, where ϵ is a small parameter defined in the next section. None of the results of the asymptotics would change with their inclusion.

Scalings

It is mathematically convenient to work in a dimensionless framework to have better scaled variables and fewer parameters. A dimensionless framework also allows "small" terms to be identified which are used in formal asymptotics to furnish highly accurate but approximate solutions. The equations presented in the main body of the paper have been converted back to dimensional form. For brevity, we present the analysis with $\bar{S} = \bar{S}^G$; the other case of $\bar{S} = \bar{S}^D$ can be handled mutatis mutandis.

We denote x for spatial position. Within Eqs. (1), (2), and (7), of the Main Manuscript, we perform a change of variables according to

$$\begin{aligned} t &= \bar{t}\tau, x = \bar{x}z \\ M(t) &= \bar{M}m(\tau), D(t) = \bar{D}d(\tau), V(t) = v(\tau) \\ S &= \bar{S}\tilde{s}, \kappa = \bar{\kappa}\tilde{\kappa}(\tau), \nu = \bar{\nu}\tilde{\nu}(\tau), \mu = \bar{\mu}\tilde{\mu}(\tau), \\ \sigma &= \bar{\sigma}\tilde{\sigma}(\tau), \end{aligned}$$

where all overlined values represent dimensional scales, and τ, m, d, v, z, along with all tilde-variables are dimensionless. We remark the rate constants are *allowed to be time-dependent*. We choose the scales $\bar{t} = \bar{\kappa}^{-1}$, $\bar{M} = \bar{S}\bar{t}$, $\bar{D} = \frac{\bar{\nu}\bar{M}^2}{\bar{\mu}}$, and $\bar{x} = \sqrt{\mathcal{D}_M \bar{t}}$. This yields the dimensionless system of equations

$$\frac{\partial m}{\partial \tau} = \Delta_z m + \tilde{s} - \tilde{\kappa} m - \nu_0(\tilde{\nu} m^2 - \tilde{\mu} d) - \epsilon^2 \zeta_0 \tilde{\nu} m d \tag{2}$$

$$\frac{\partial d}{\partial \tau} = \xi^2 \Delta_z d + \epsilon^{-1} \mu_0 (\tilde{\nu} m^2 - \tilde{\mu} d) - \frac{1}{2} \nu_0 \tilde{\nu} m d \tag{3}$$

$$\frac{\mathrm{d}v}{\mathrm{d}\tau} = -\epsilon^2 \sigma_0 \tilde{\sigma} d v \tag{4}$$

with values appearing in Table B. The use of $0 < \epsilon \ll 1$ is suggestive of formal asymptotics.

ODEs

We first consider equations Eqs. (2)-(4) in the absence of diffusion so that the equations are ordinary differential equations and we solve them subject to $m(\tau = 0) = d(\tau = 0) = 0$, with an initial concentration of monomers and dimers of zero, and with $v(\tau = 0) = 1$, i.e., the cell viability is initially at its maximum. We begin by finding asymptotic solutions for m and d and deal with v later.

Parameter	Definition	Value
$\boldsymbol{\epsilon}$	$\frac{\bar{\sigma}^{1/3}\bar{D}^{1/3}}{\bar{\mu}^{1/3}}$	8.85×10^{-4}
$\boldsymbol{\nu_0}$	$\frac{2\bar{\nu}\bar{S}}{\bar{\kappa}^2}$	0.220
$\boldsymbol{\zeta_0}$	$\frac{\lambda_k^{2/3}\bar{\nu}^2\bar{S}^2}{\bar{\mu}^{1/3}\bar{\kappa}^3}$	2.39
$\boldsymbol{\mu_0}$	$\frac{\bar{\mu}^{2/3}}{\bar{\kappa}\lambda_\kappa^{1/3}}$	5.75
$\boldsymbol{\sigma_0}$	$\frac{\bar{\sigma}\bar{\nu}\bar{S}^2\lambda_\kappa^{2/3}}{\bar{\mu}^{1/3}\bar{\kappa}^3}$	0.102
$\boldsymbol{\xi}$	$\sqrt{\frac{\mathcal{D}_D}{\mathcal{D}_M}}$	0.887
$\boldsymbol{\lambda_{0\kappa}}$	$\frac{\lambda_\kappa^{1/3}\bar{\kappa}}{\bar{\mu}^{2/3}}$	0.174
$\boldsymbol{\lambda_{0S,G}}$	$\frac{\lambda_S^G\bar{\kappa}}{\lambda_\kappa^{2/3}\bar{\mu}^{2/3}}$	0.234
$\boldsymbol{\lambda_{0S,D}}$	$\frac{\lambda_S^D\bar{\kappa}}{\lambda_\kappa^{2/3}\bar{\mu}^{2/3}}$	0.131

Table B. Dimensionless parameters. With $\epsilon \ll 1$ chosen, these serve as constants for the asymptotic calculations. For the values displayed, we use $\bar{S} = \bar{S}^G$. All parameters except for ϵ are $O(1)$. The bottom parameters ensure that the slow timescales over which κ and S change are on the scale of $1/\epsilon^2$.

Since the monomer clearance rate and activity of the $\beta-$secretase varies over decades (where $\tau = O(1/\epsilon^2)$), assume a similar behavior in other rate constants and model this by

$$\begin{aligned}
\tilde{s}(\tau) &= \tilde{s}_s(\epsilon^2\tau) = \tilde{s}_s^*(\epsilon^2\tau/\lambda_{0S}) \\
\tilde{\kappa}(\tau) &= \tilde{\kappa}_s(\epsilon^2\tau) = \tilde{\kappa}_s^*(\epsilon^2\tau/\lambda_{0\kappa}) \\
\tilde{\nu}(\tau) &= \tilde{\nu}_s(\epsilon^2\tau) \\
\tilde{\mu}(\tau) &= \tilde{\mu}_s(\epsilon^2\tau) \\
\tilde{\sigma}(\tau) &= \tilde{\sigma}_s(\epsilon^2\tau)
\end{aligned}$$

for $O(1)$ functions with $O(1)$ rates of change $\tilde{s}_s, \tilde{\nu}_s, \tilde{\mu}_s$, $\tilde{\sigma}_s$, and $\tilde{s}_s^*(\tilde{\tau}) = 1 + \tilde{\tau}$. The functions $\tilde{\kappa}_s$ and $\tilde{\kappa}_s^*$ with $\tilde{\kappa}_s^*(\tilde{\tau}) = 1 - \tilde{\tau}$ are $O(1)$ with $O(1)$ rates of change but may become $o(1)$ as the argument of $\tilde{\kappa}_s^*$ approaches 1. We assume all the rates change over a slow, $O(1/\epsilon^2)$ timescale. The subscripts in functions such as κ_s signify a *slowly evolving* function. The constants $\lambda_{0S,G}$, $\lambda_{0S,D}$, and $\lambda_{0\kappa}$ are $O(1)$ with $\lambda_{0S,G}$ used for the general population and $\lambda_{0S,G}$ used for the Down Syndrome population — values appear in Table B. As we have approximate forms for how S and κ vary, we are more precise about the form their dimensionless representations take.

The system admits multiple time scales but our approach is to treat the system as a set of inner-outer matching problems. Over a very fast time scale (indicated by a subscript f), $\tau_f = \tau/\epsilon$, and with $m \sim \epsilon m^{(f)}, d \sim \epsilon^2 d^{(f)}$ for $O(1)$, the ODEs are

$$\begin{aligned}
m_{\tau_f}^{(f)} &= \tilde{s}_s(0) \\
d_{\tau_f}^{(f)} &= \tilde{\nu}_s(0){m^{(f)}}^2 - \tilde{\mu}_s(0)d^{(f)}
\end{aligned}$$

so that from our initial conditions, $m^{(f)}(0) = d^{(f)}(0) = 0$,

$$\begin{aligned}
m^{(f)}(\tau_f) &= \tilde{s}_s(0)\tau_f \\
d^{(f)}(\tau_f) &= \frac{\tilde{s}_s^2(0)\tilde{\nu}_s(0)\tau_f^2}{\tilde{\mu}_s(0)} - \frac{\tilde{s}_s^2(0)\tilde{\nu}_s(0)\tau_f}{\tilde{\mu}_s^2(0)} - \frac{2\tilde{s}_s^2(0)\tilde{\nu}_s(0)\mathrm{e}^{-\tilde{\mu}_s(0)\tau_f}}{\tilde{\mu}_s^3(0)} + \frac{2\tilde{s}_s^2(0)\tilde{\nu}_s(0)}{\tilde{\mu}_s^3(0)}
\end{aligned}$$

Over the "normal" timescale we posit that $m \sim m^{(n)}$ and $d \sim d^{(n)}$ for $O(1)$ functions $m^{(n)}$ and $d^{(n)}$ to obtain

$$m^{(n)}_\tau = \tilde{s}_s(0) - \tilde{\kappa}_s(0) m^{(n)}$$
$$0 = \tilde{\nu}_s(0) {m^{(n)}}^2 - \tilde{\mu}_s(0) d^{(n)}$$

which can be trivially matched to the innermost τ_f-region with

$$m^{(n)}(\tau) = \frac{\tilde{s}_s(0)}{\tilde{\kappa}_s(0)}(1 - \mathrm{e}^{-\tilde{\kappa}_s(0)\tau}) \tag{5}$$
$$d^{(n)}(\tau) = \frac{\tilde{\nu}_s(0)}{\tilde{\mu}_s(0)} m_0^2 \tag{6}$$

In order to observe the system response to changing rate constants, we consider a slow timescale $\tau_s = \epsilon^2 \tau$. Because $\tilde{\kappa}^*(\tilde{\tau}) = 0$ at $\tilde{\tau} = 1$, for our analysis, we assume that $1 - \tau_s/\lambda_{0\kappa} = O(1)$. Here, we take $m \sim m^{(s)}$ and $d \sim d^{(s)}$ for $O(1)$ solutions $m^{(s)}$ and $d^{(s)}$. In this case,

$$0 = \tilde{s}_s(\tau_s) - \tilde{\kappa}_s(\tau_s) m_0$$
$$0 = \tilde{\nu}(\tau_s) m_0^2 - \tilde{\mu}(\tau_s) d_0$$

The two equations do not have explicit τ_s-derivatives: they are a system of algebraic equations that come about through the long-time evolution of Eqs. (5)-(6). This gives

$$m^{(s)}(\tau_s) = \frac{\tilde{s}_s(\tau_s)}{\tilde{\kappa}_s(\tau_s)} \tag{7}$$
$$d^{(s)}(\tau_s) = \frac{\tilde{\nu}_s(\tau_s)\tilde{s}_s^2(\tau_s)}{\tilde{\mu}_s(\tau_s)\tilde{\kappa}_s^2(\tau_s)} \tag{8}$$

A composite solution on $\tau \lesssim 1/\epsilon^2$ with $1 - \epsilon^2\tau/\lambda_{0\kappa}$ being $O(1)$ can be obtained as

$$m \sim m_0 := \frac{\tilde{s}(\tau)}{\tilde{\kappa}(\tau)} - \frac{\tilde{s}(0)}{\tilde{\kappa}(0)}\mathrm{e}^{-\tilde{\kappa}(0)\tau} \tag{9}$$
$$d \sim d_0 := \frac{\tilde{\nu}(\tau)}{\tilde{\mu}(\tau)}\Big(\frac{\tilde{s}(\tau)}{\tilde{\kappa}(\tau)} - \frac{\tilde{s}(0)}{\tilde{\kappa}(0)}\mathrm{e}^{-\tilde{\kappa}(0)\tau}\Big)^2 \tag{10}$$

We shall refer to these leading order solutions as m_0 and d_0, respectively.
From (4), we have that

$$v(\tau) = \exp\Big(-\epsilon^2\sigma_0 \int_0^\tau \tilde{\sigma}(u)(d_0(u) + o(1))\mathrm{d}u\Big)$$

This can be evaluated to

$$v(\tau) = \begin{cases} 1 - \epsilon^2\sigma_0 \frac{\tilde{\sigma}(0)\tilde{\nu}(0)}{\tilde{\mu}(0)}\Big(\frac{\tilde{s}(0)^2}{\tilde{\kappa}(0)^2}\tau + \frac{2\tilde{s}(0)}{\tilde{\kappa}(0)^2}(\mathrm{e}^{-\tilde{\kappa}(0)\tau} - 1) \\ \quad + \frac{1}{2\tilde{\kappa}(0)}(\mathrm{e}^{-2\tilde{\kappa}(0)\tau} - 1)\Big) + o(\epsilon^2), \quad \tau \ll O(1/\epsilon^2) \\ \exp\Big(-\sigma_0 \int_0^{\epsilon^2\tau} \frac{\tilde{\sigma}_s(u)\tilde{\nu}_s(u)\tilde{s}_s^2(u)}{\tilde{\mu}_s(u)\tilde{\kappa}_s^2(u)}\mathrm{d}u\Big) + o(1), \quad \tau = O(1/\epsilon^2), 1 - \epsilon^2\tau/\lambda_{0\kappa} = O(1) \end{cases} \tag{11}$$

We focus on an $O(1)$ description of v so that a uniformly valid approximation for $\tau \lessapprox 1/\epsilon^2$ and $1 - \epsilon^2\tau/\lambda_{0\kappa} = O(1)$ is

$$v \sim \exp\Big(-\epsilon^2\sigma_0 \int_0^\tau \frac{\tilde{\sigma}(u)\tilde{\nu}(u)\tilde{s}^2(u)}{\tilde{\mu}(u)\tilde{\kappa}^2(u)}\mathrm{d}u\Big). \tag{12}$$

The solutions presented in the main body of the paper are for the biologically relevant timescale of decades, i.e., $\tau = O(1/\epsilon^2)$ with $1 - \epsilon^2\tau/\lambda_{0\kappa} = O(1)$.

As a mathematical remark, when $1 - \epsilon^2\tau/\lambda_{0\kappa} = O(\epsilon)$, a different asymptotic regime is obtained. However, this analysis is not relevant to the problem at hand and we do not analyze it.

PDEs

In analyzing the PDE models, there are a number of asymptotic balances that are possible, i.e., depending on the spatial, temporal, or concentration scales that we look at, different terms in the equations dominate the system behavior. We study one of the most biologically relevant balances.

Throughout this analysis, we will focus upon the local effects of a perturbation in the monomer production. We consider a radially symmetric perturbation so that spherical symmetry and various simplifications can be applied. The solutions presented are obtained from the Green's functions provided later.

Spatial Model Results

We posit $z, \tau = O(1)$, $m \sim m_0 = O(1)$, $d \sim d_0 = O(1)$, and $\tilde{s} = O(1)$. Biologically, this describes the system on a length-scale of $\bar{X}$ and a timescale of $\bar{T}$, with the monomers and dimers being on their characteristic scales $\bar{M}$ and $\bar{D}$. Denoting $r = |z|$ with the centre of the disturbance at $r = 0$, we have

$$\begin{aligned} m_{0,\tau} &= m_{0,rr} + \frac{2}{r} m_{0,r} + \tilde{s} - \tilde{\kappa} m_0 \\ 0 &= \tilde{\nu} m_0^2 - \tilde{\mu} d_0. \end{aligned}$$

If $\tilde{s} = \tilde{s}_0 + \rho \begin{cases} 1, & r < R \\ 0, & r \geq R \end{cases}$ where R denotes the dimensionless radius of a hypothetical sphere where monomer production is increased, then using (22) and (21) with $\alpha = 1$, $\beta = \tilde{\kappa}$, $\phi = \tilde{s}$, we can explicitly solve for m_0 at steady state giving

$$m_0(r) = \frac{\tilde{s}_0}{\tilde{\kappa}} + \rho \begin{cases} \frac{\mathrm{e}^{-\sqrt{\tilde{\kappa}}r}}{\sqrt{\tilde{\kappa}}r} \left[\frac{r}{\sqrt{\tilde{\kappa}}} \cosh(\sqrt{\tilde{\kappa}}r) - \frac{1}{\tilde{\kappa}} \sinh(\sqrt{\tilde{\kappa}}r) \right] \\ + \frac{\sinh(\sqrt{\tilde{\kappa}}r)}{\sqrt{\tilde{\kappa}}r} \left[\frac{r\mathrm{e}^{-\sqrt{\tilde{\kappa}}r}}{\sqrt{\tilde{\kappa}}} - \frac{R\mathrm{e}^{-\sqrt{\tilde{\kappa}}R}}{\sqrt{\tilde{\kappa}}} \right. \\ \left. - \frac{1}{\tilde{\kappa}} \mathrm{e}^{-\sqrt{\tilde{\kappa}}R} + \frac{1}{\tilde{\kappa}} \mathrm{e}^{-\sqrt{\tilde{\kappa}}r} \right], & r < R \\ \frac{\mathrm{e}^{-\sqrt{\tilde{\kappa}}r}}{\sqrt{\tilde{\kappa}}r} \left[\frac{R}{\sqrt{\tilde{\kappa}}} \cosh(\sqrt{\tilde{\kappa}}R) - \frac{1}{\tilde{\kappa}} \sinh(\sqrt{\tilde{\kappa}}R) \right], \\ \quad r \geq R, \end{cases} \tag{13}$$

and

$$d_0 = \frac{\tilde{\nu}}{\tilde{\mu}} m_0^2. \tag{14}$$

The fact that we only consider the steady-state here is motivated by our observation in the ODE model that over an $O(1)$ time in τ, the viability loss is $O(\epsilon^2)$, which is negligible. The most important dynamics occur over the τ_s timescale whereby m_0 and d_0 can be taken as steady-state values.

Solutions via Green's Functions

The preceding solutions were obtained using Green's functions. In general, to solve the spherically symmetric problem

$$h_t = \alpha(h_{rr} + \frac{2}{r}h_r) - \beta h + \phi(r,t) \tag{15}$$

$$h(r,0) = h_0(r) \tag{16}$$

$$h(\infty,t) \text{ is bounded}, \quad h_r(0,t) = 0 \tag{17}$$

for $h(r,t)$, we find that

$$h(r,t) = \int_0^\infty \int_0^\infty {r^*}^2 \phi(r^*,t^*) G(r^*,t^*;r,t) \mathrm{d}r^* \mathrm{d}t^* \tag{18}$$

$$+ \int_0^\infty {r^*}^2 h_0(r^*) G(r^*,0;r,t) \mathrm{d}r^* \tag{19}$$

where

$$G(r^*,t^*;r,t) = \frac{\Theta(t-t^*)}{2\sqrt{\pi\alpha(t-t^*)}rr^*}\Big(\mathrm{e}^{-\frac{(r-r^*)^2}{4\alpha(t-t^*)}} - e^{-\frac{(r+r^*)^2}{4\alpha(t-t^*)}}\Big)\mathrm{e}^{-\beta(t-t^*)}. \tag{20}$$

The function Θ denotes the Heaviside step function,

$$\Theta(x) = \begin{cases} 1, & x \geq 0 \\ 1/2, & x = 0 \\ 0, & x < 0. \end{cases}$$

Also, if ϕ does not depend on time, the steady-state solution can be found from

$$h(r) = \int_0^\infty {r^*}^2 G(r^*;r)\phi(r^*)\mathrm{d}r^* \tag{21}$$

with G now given by

$$G(r^*;r) = \begin{cases} \frac{1}{2\sqrt{\alpha\beta}rr^*}(\mathrm{e}^{-\sqrt{\beta/\alpha}(r-r^*)} - \mathrm{e}^{-\sqrt{\beta/\alpha}(r+r^*)}), \\ r^* < r \\ \frac{1}{2\sqrt{\alpha\beta}rr^*}(\mathrm{e}^{-\sqrt{\beta/\alpha}(r^*-r)} - \mathrm{e}^{-\sqrt{\beta/\alpha}(r+r^*)}), \\ r^* \geq r. \end{cases} \tag{22}$$

Asymptotic Validity

There are precise ranges over which the asymptotic results will be valid. Taking the system out of the well-defined scalings will result in approximations that become less valid and could ultimately fail. We summarize here the important considerations in using these formulas.

ODEs

Some essential conditions are listed below:

1. The value ϵ defined in Table B is *small*, i.e. much smaller than 1.
2. *With respect to the* ϵ parameter, the following values can be said to be $O(1)$, i.e., much bigger than ϵ and much smaller than $1/\epsilon$: ν_0, ζ_0, σ_0, ξ, $\lambda_{0\kappa}$, $\lambda_{0S,G}$, and $\lambda_{0S,D}$.

3. If the equations are evolved for a period of time T_{evolve} then over that time, $\tilde{s}(t/\bar{t})$, $\tilde{\kappa}(t/\bar{t})$, $\tilde{\mu}(t/\bar{t})$, $\tilde{\nu}(t/\bar{t})$, and $\tilde{\sigma}(t/\bar{t})$ are $O(1)$ for $0 \le t \le T_{\text{evolve}}$.

We remark that no conditions are placed upon γ.

PDEs

For the PDE analysis, we presented the solution at *steady state* and the rate parameters should not be changing. The solution is valid provided items 1 and 2 of the ODE conditions above hold and the spatial and temporal scales are $O(1)$.

Uncertainty Quantification and Damage Distributions

Notation

Here we adopt some notational conventions and assumptions. We shall denote

$$U(t) = U_0 \Xi_U(t) \tag{23}$$
$$\omega(t) = \omega_0 \Xi_\omega(t) \tag{24}$$

to be the (possibly) time-dependent cell damage rate and AD development rate where $U(0) = U_0$, $\omega(0) = \omega_0$, and $\Xi_U(t)$ and $\Xi_\omega(t)$ are scaling functions that combine the effects of aging and lifestyle factors.

We will generally consider that U_0 is either fixed or that it has a distribution within the population, likewise for ω_0. But for simplicity, we assume that within a population, $\Xi_U(t)$ and $\Xi_\omega(t)$ are the same for all people. In the static model, $\Xi_U = \Xi_\omega = 1$ but in the dynamic model, both Ξ_U and Ξ_ω increase. Using only how $\kappa(t)$ and $S(t)$ vary, we have that $\Xi_U(t) = \Xi_\omega(t) = \left(\frac{1+t/\lambda_S}{1-t/\lambda_\kappa}\right)^2$. The distinction between Ξ_U and Ξ_ω is not necessary in the current model. But in a more general setting where possibly γ is time-dependent, the functions will not be the same.

We denote the integration operator

$$\mathcal{I}_{t_1,t_2}[\cdot] = \int_{t_1}^{t_2} (\cdot) \mathrm{d}s. \tag{25}$$

We denote g_U to be a probability density for U_0. And we denote g_ω to be a probability density for ω_0. When U_0 and ω_0 are discrete, the densities are understood to allow for $\delta-$function so that integration represents a sum. In reality g_U and g_ω are probability measures. Integrations with respect to probability measures are understood to range over the entire set of values for the corresponding random variable.

We shall denote $g_U(u) = \Pr(U_0 = u)$ with the understanding that if g is a density then $g(u)\mathrm{d}u = \Pr(u \le U_0 < u + \mathrm{d}u)$ for a differential $\mathrm{d}u$. We adopt similar notation with $g_\omega(w) = \Pr(\omega_0 = w)$. We furthermore denote $g_U(u|E)$ as the probability density for $U_0 = u$ conditioned on the event E and likewise for $g_w(w|E)$. Through Bayes theorem, we have

$$g_U(u|E) = \frac{\Pr(E|U_0 = u) g_U(u)}{\int \Pr(E|U_0 = u) \mathrm{d}g_U(u)}$$
$$g_\omega(w|E) = \frac{\Pr(E|\omega_0 = w) g_\omega(w)}{\int \Pr(E|\omega_0 = w) \mathrm{d}g_\omega(w)}.$$

General Hazard Function

Let T be a random variable denoting the time of an event (such as AD development or the death of a neuron). Let $\Gamma(t)$ be the Hazard function for the time of an event. Let $\mathrm{d}t$ denote an infinitesimal time interval. Then, by definition,

$$\Pr(t < T \leq t + \mathrm{d}t | T > t) = \Gamma(t)\mathrm{d}t.$$

The probability density for T, call it $f(t)$ satisfies

$$f(t)\mathrm{d}t = \Pr(t < T \leq t + \mathrm{d}t).$$

Thus, by the definition of conditional probability,

$$\Gamma(t)\mathrm{d}t = \frac{\Pr(t < T \leq t + \mathrm{d}t \wedge T > t)}{\Pr(T > t)} = \frac{f(t)\mathrm{d}t}{\Pr(T > t)}.$$

We conclude that

$$f(t) = \Pr(T > t)\Gamma(t).$$

Let $\hat{f}(t) = \int_0^t f(s)\mathrm{d}s$ be the cumulative distribution function for T. Then $\hat{f}'(t) = (1 - \hat{f}(t))\Gamma(t)$ so that

$$\hat{f}(t) = 1 - \exp(-\int_0^t \Gamma(s)\mathrm{d}s)$$

and

$$f(t) = \Gamma(t)\exp(-\int_0^t \Gamma(s)\mathrm{d}s).$$

Thus, given a hazard function, the probability density function can be computed. And

$$\Pr(T \leq t) = \hat{f}(t) = 1 - \exp(-\int_0^t \Gamma(s)\mathrm{d}s). \tag{26}$$

Note that if $t(\sup_{0 \leq s \leq t} \Gamma(s)) \ll 1$, then $\Pr(T \leq t) \approx \int_0^t \Gamma(s)\mathrm{d}s$ by a Taylor expansion.

Viability

Recall that we denote $V(t)$ to be the fractional density of healthy neurons relative to their initial healthy value. Let N_0 be the initial number of healthy neurons in a small volume with $N(t)$ being the number of viable neurons in that volume a time t later. For each neuron, let T be time of death for each neuron with hazard function $U_0\Xi(t)$. Then the probability density function (pdf) for the time of death is

$$f(t) = U_0\Xi_U(t)\exp(-U_0\mathcal{I}_{0,t}\Xi_U).$$

Then we have immediately that $\Pr(T > t) = \exp(-U_0\mathcal{I}_{0,t}\Xi_U)$ from (26) with $\Gamma = U_0\Xi_U$. Thus, at time t, the number of viable neurons, $N(t)$, is a Binomial random variable with

$$\mathbb{E}[V(t)] = \mathbb{E}[\frac{N(t)}{N_0}] = \exp(-U_0\mathcal{I}_{0,t}\Xi_U)$$
$$\mathrm{Var}[V(t)] = \mathrm{Var}[\frac{N(t)}{N_0}] = \frac{\exp(-U_0\mathcal{I}_{0,t}\Xi_U)(1 - \exp(-U_0\mathcal{I}_{0,t}\Xi_U))}{N_0}.$$

As $N_0 \uparrow \infty$, to describe a large number of neurons, the variance $\downarrow 0$. Thus, the mean and all percentiles converge to $\exp(-U_0 \int_0^t \Xi_U(s)\mathrm{d}s)$. We therefore use this as the value of $V(t)$ for anyone with neuronal damage rate U_0.

Prevalence and Incidence

For someone to have AD at age t, they must have been diagnosed later than $t - T_D$ because our model assumes death occurs T_D years after diagnosis. Thus, the prevalence of AD at age t is the number of people diagnosed later than $t - T_D$ who were diagnosed on the interval $(t - T_D, t]$ divided by the number diagnosed later than $t - T_D$. In all of this there is an implicit assumption that the individuals did not die from other causes by age t. For simplicity, we assume all other causes of death act independent of AD.

Within the general population, the hazard function of AD development is $\omega_0 \Xi_\omega$.

We denote $\mathcal{A}(a, b)$ to be event that an individual is diagnosed with AD on the time interval $(a, b]$. Let $N_0(t)$ here denote the number of people diagnosed later than t. Let $p_{\tilde{t}}(t)$ denote $\Pr(\mathcal{A}(t - \tilde{t}, t) | \mathcal{A}(t - \tilde{t}, \infty))$. Performing the calculation, we have

$$\begin{aligned} p_{\tilde{t}}(t) &= \frac{\Pr(\mathcal{A}(t - \tilde{t}, t))}{\Pr(\mathcal{A}(t - \tilde{t}, \infty))} \\ &= \frac{\exp(-\omega_0 \mathcal{I}_{0,t-\tilde{t}} \Xi_\omega) - \exp(-\omega_0 \mathcal{I}_{0,\tilde{t}} \Xi_\omega)}{\exp(-\omega_0 \mathcal{I}_{0,t-\tilde{t}} \Xi_\omega)} \\ &= 1 - \exp(-\omega_0 \mathcal{I}_{t-\tilde{t},t} \Xi_\omega). \end{aligned} \tag{27}$$

Let $N_P(t)$ denote the number of people diagnosed on $(t - T_D, t]$. Then $P(t) = \frac{N_P(t)}{N_0(t - T_D)}$ so that

$$\mathbb{E}[P(t)] = p_{T_D}(t) \tag{28}$$

$$\mathrm{Var}[P(t)] = \frac{p_{T_D}(t)(1 - p_{T_D}(t))}{N_0(t - T_D)}. \tag{29}$$

We observe that $p_{\tilde{t}}(t) = 1 - \exp(-\omega_0 \mathcal{I}_{t-\tilde{t},t} \Xi_\omega) = \omega_0 \Xi_\omega(t)\tilde{t} + o(\omega_0 \Xi_\omega(t)\tilde{t})$.

The incidence, for an infinitesimal time step $\mathrm{d}t$, is the fraction of people diagnosed on $(t - \mathrm{d}t, t]$ divided by $\mathrm{d}t$. Let $N_{I,\Delta t}$ denote the number diagnosed on $(t - \Delta t, t]$. Then $I(t) = \frac{N_{I,\Delta t}}{\Delta t N_0(t - \Delta t)}$. Thus letting $\Delta t \downarrow 0$:

$$\mathbb{E}[I(t)] = \omega_0 \Xi_\omega(t) \tag{30}$$

$$\mathrm{Var}[I(t)] = \frac{\omega_0 \Xi(t)}{\Delta t N_0(t)}. \tag{31}$$

The Central Limit Theorem tells us that $P(t)$ and $I(t)$, for large enough populations, will be normally distributed according to
$\mathcal{N}(1 - \exp(-\omega_0 \mathcal{I}_{t-T_D,t} \Xi_\omega), \frac{(1-\exp(-\mathcal{I}_{t-T_D,t}\Xi_\omega))\exp(-\mathcal{I}_{t-T_D,t}\Xi_\omega)}{N_0(t-T_D)})$ and $\mathcal{N}(\omega_0 \Xi(t), \frac{\omega_0 \Xi(t)}{\Delta t N_0(t)})$, respectively. If Δt is truly taken to 0, the variance of the incidence diverges. In practice, observations are done over small, but finite time windows.

The mean values are precisely those in Eqs. $(8)_2$ and $(9)_2$ of the Main Manuscript. Furthermore, we see that for large enough populations, all the percentiles converge to the mean values. As AD data are observed over very large population sizes, we do not plot error bars in our model predictions.

In general, with a finite initial population of $N_0(0)$, we can estimate the 95%-confidence interval for a population under observation as *approximately* within 1.96 standard deviations of the mean, where the standard deviation is inversely proportional to the square root of $N_0(0)$. In (29) and (31), the denominator is time-dependent, however, so once people have died from AD, this approximation will begin to lose accuracy.

As later depicted in Fig C, the 95% confidence window does observe this scaling. For instance, at age 60, the dynamic model with fixed $\omega(t) = \omega_0 \Xi_\omega(t)$ having a single value $\omega_0 = \bar{\omega}$ and with an age-related scaling, we find the 95% confidence windows for prevalence and incidence are $0.430 - 0.563\%$ and $0.0352 - 0.127\%$/y, respectively. With $N_0 = 40,000$ treated as constant and with $\Delta t = 0.25$ y, Eqs. (29) and (31) predict widths of the 95% confidence windows of 0.133% and 0.0918%/yr, respectively. This is completely consistent with the observed intervals of 0.136% and 0.111%/yr, respectively.

Another thing to note is that at leading order, $P(t) = \omega_0 \int_{t-T_D}^{t} \Xi_\omega(s)\mathrm{d}s$, which is proportional to ω_0. Thus, if monomer production $S(t)$ increased by a factor Ω, prevalence would increase by a factor of $\approx \Omega^2$. Also, the doubling times for incidence and prevalence, when fitted to exponential growth, do not depend upon ω_0.

Lifetime Risk

The pdf for diagnosis at age t is given by $\omega_0 \Xi_\omega(t) \exp(-\omega_0 \int_0^t \Xi_\omega(s)\mathrm{d}s)$ and thus, by integrating from $t = 0$ to $t = T$, we can compute the cumulative risk up to age T.

$$\Upsilon(T) = 1 - \exp(-\omega_0 \int_0^T \Xi_\omega(s)\mathrm{d}s).$$

We can also compute the risk of developing AD over an interval t_1 to t_2 given it has not occurred up to t_1 via

$$1 - \exp(-\omega_0 \int_{t_1}^{t_2} \Xi_\omega(s)\mathrm{d}s).$$

Average Initial Neuronal Damage Rate U_0 within AD Patients

We consider the possibility that U_0 has a distribution of values within the population. We suppose that $U_0 \sim g_U(u)$. We suppose further that $\mathbb{E}[U_0] = U^*$ is the mean value and $\mathrm{Var}[U_0] = \Sigma^{*2}$ is the variance. To exploit various asymptotic approximations, we operate under the assumptions:

- $\Xi_U(t) = O(1)$ over the desired time span,
- $U^* \sim \Sigma^*$ in the sense they are in the same ballpark of values,
- $\gamma T_D U^*, \gamma T_D \Sigma^* \ll 1$, and
- $g_U(u)$ is exponentially small when $\gamma T_D u \geq O(1)$.

In practice many distributions fit this requirement. Note also that $\mathcal{I}_{t-T_D,t}\Xi_U = O(T_D)$.

We wish to understand if the average value of U_0 among those with AD is higher than U^*, i.e., *if those with AD may have a larger rate of neuronal loss, on average.* To this end, we seek the density

$$g_U(u|\text{AD at age } t) = \frac{\Pr(\text{AD at age } t|U_0 = u) g_U(u)}{\int \Pr(\text{ AD at age } t|U_0 = u)\mathrm{d}g_U(u)}.$$

Colloquially, it is the density for U_0 given that someone has AD. We wish to understand if

$$\mathbb{E}(U_0|\text{AD}^+) = \int_0^\infty u \mathrm{d}g_U(u|\text{AD at age } t)$$

is larger than U^*, the mean value of U_0.

$\Pr(\text{AD at age } t|U_0 = u) = 1 - \exp(\gamma u \mathcal{I}_{t-T_D,t}\Xi_U)$ is the prevalence of AD with $\bar{U} = u$, which, by Taylor series, is approximately $\gamma u \mathcal{I}_{t-T_D,t}\Xi_U$, for small enough $\gamma T_D u$.

Also, by compliment,
$\Pr(\text{no AD at age } t|U_0 = u) = \exp(\gamma u \mathcal{I}_{t-T_D,t}\Xi_U) \approx 1 - \gamma u \mathcal{I}_{t-T_D,t}\Xi_U$. Note that to not have AD at age t implicitly means being alive at that age, so the same conditional requirement that diagnosis takes place after $t - T_D$ is present.

Thus, with formal asymptotics,

$$\mathbb{E}(U_0|\text{AD}^+) \sim \int_0^\infty \frac{\gamma u^2 \mathcal{I}_{t-T_D,t}\Xi_U}{\int_0^\infty \gamma w \mathcal{I}_{t-T_D,t}\Xi_U \mathrm{d}g_U(u')} \mathrm{d}g_U(u) = \frac{1}{\mathbb{E}(U_0)} \int_0^\infty u^2 \mathrm{d}g_U(u)$$
$$= \frac{\mathbb{E}(U_0^2)}{\mathbb{E}(U_0)} = U^* + \frac{\Sigma^{*2}}{U^*} > U^*.$$

We have used $\text{Var}(\cdot) = \mathbb{E}(\cdot^2) - \mathbb{E}(\cdot)^2$.

We can also compute the expected value of U_0 given someone does not have AD. Using similar notation and approximations, we have

$$g_U(u|\text{no AD at age } t) = \frac{\Pr(\text{no AD at age } t|U_0 = u)}{\int_0^\infty \Pr(\text{no AD at age } t|U_0 = u)\mathrm{d}g_U(u)}$$

with

$$\begin{aligned}
\mathbb{E}(U_0|\text{AD}^-) &\sim \int_0^\infty \frac{u(1 - \gamma u \mathcal{I}_{t-T_D,t}\Xi_U)}{\int_0^\infty (1 - \gamma u' \int_{t-T_D}^t \Xi_U)\mathrm{d}g_U(u')} \mathrm{d}g_U(u) \\
&= \frac{\mathbb{E}(U_0) - \gamma \mathcal{I}_{t-T_D,t}\Xi_U \mathbb{E}(\bar{U}_0^2)}{1 - \gamma \mathcal{I}_{t-T_D,t}\Xi_U \mathbb{E}(\bar{U})} \\
&\sim U^* - \gamma \mathcal{I}_{t-T_D,t}\Xi_U \Sigma^{*2} + O(\gamma^2 T_D^2 U^{*2}).
\end{aligned}$$

To leading order, this is U^*.

As a check, we have that

$$\begin{aligned}
\mathbb{E}(\bar{U}) &= \mathbb{E}(\bar{U}|\text{AD}^+)\Pr(\text{AD}^+) + \mathbb{E}(\bar{U}|\text{AD}^-)\Pr(\text{AD}^-) \\
&\sim (U^* + \frac{\Sigma^{*2}}{U^*}) \int_0^\infty g(u)\gamma u \mathcal{I}_{t-T_D,t}\Xi_U \\
&\quad + (U^* - \gamma \mathcal{I}_{t-T_D,t}\Xi_U \Sigma^{*2}) \times \\
&\int_0^\infty g(u)(1 - \gamma u \mathcal{I}_{t-T_D,t}\Xi_U)\mathrm{d}u \\
&= (U^* + \frac{\Sigma^{*2}}{U^*})\gamma U^* \mathcal{I}_{t-T_D,t}\Xi_U \\
&+ (U^* - \gamma \mathcal{I}_{t-T_D,t}\Xi_U \Sigma^{*2})(1 - \gamma \bar{U} \mathcal{I}_{t-T_D,t}\Xi_U) \\
&= U^*(1 + O(\gamma^2 \Sigma^{*2} T_D^2))
\end{aligned}$$

We can see these approximations are accurate in Fig B.

Effects of Distribution of AD Development Rate ω_0

Here we consider calculating the prevalence and incidence with a distribution of ω_0-values in the population. Here g_ω is now describing the density for ω_0.

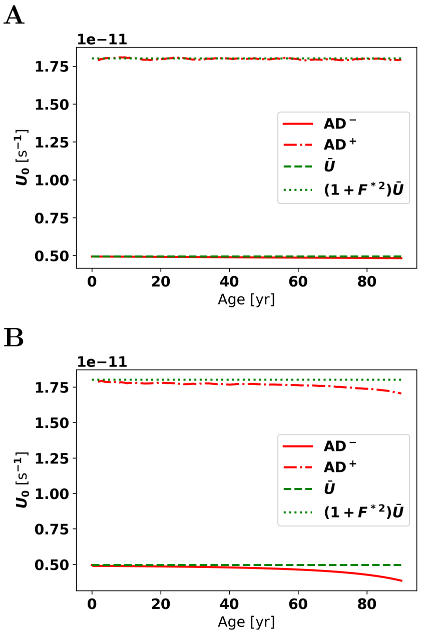

Fig B. U_0 values conditioned on disease. The mean value of U_0 is plotted among patients with AD, AD^+ and patients without AD, AD^- as a function of age. The approximations derived above are extremely accurate, even in the dynamic model. *A:* static model. *B:* dynamic model.

$$
\begin{aligned}
&\Pr(\mathcal{A}(t-\tilde{t},t)|\mathcal{A}(t-\tilde{t},\infty)) \\
&= \int \Pr(\mathcal{A}(t-\tilde{t},t)|\omega_0 = w \wedge \mathcal{A}(t-\tilde{t},\infty))\mathrm{d}g_\omega(w|\mathcal{A}(t-\tilde{t},\infty)) \\
&= \int (1-\mathrm{e}^{-w\int_{t-\tilde{t}}^{t}\Xi(s)\mathrm{d}s}) \frac{\Pr(\mathcal{A}(t-\tilde{t},\infty)|\omega_0 = w)\mathrm{d}g_\omega(w)}{\int \Pr(\mathcal{A}(t-\tilde{t},\infty)|\omega_0 = w')\mathrm{d}g_\omega(w')} \implies \\
&= \frac{\int (1-\mathrm{e}^{-w\int_{t-\tilde{t}}^{t}\Xi(s)\mathrm{d}s})\mathrm{e}^{-w\int_0^{t-\tilde{t}}\Xi(s)\mathrm{d}s}\mathrm{d}g_\omega(w)}{\int \mathrm{e}^{-w'\int_0^{t-\tilde{t}}\Xi(s)\mathrm{d}s}\mathrm{d}g_\omega(w')}.
\end{aligned}
$$

This allows for two important calculations. The prevalence is defined by $\Pr(\mathcal{A}(t-T_D,t)|\mathcal{A}(t-T_D,\infty))$ and the incidence is found by $\lim_{\tilde{t}\downarrow 0}\frac{1}{\tilde{t}}\Pr(\mathcal{A}(t-\tilde{t},t)|\mathcal{A}(t-\tilde{t},\infty))$. In particular, with the parameters of the example distribution of Table 2 of the Main Manuscript, we have $U^- = \bar{U}/10$ with $p^- = 0.765$ and $U^+ = 3.93\bar{U}$ with $p^+ = 1-p^- = 0.235$. Let $\omega^- = \gamma U^-$ and $\omega^+ = \gamma U^+$. The results are

$$
\begin{aligned}
P(t) = &\left(p^-\mathrm{e}^{-\omega^-\int_0^{t-T_D}\Xi\mathrm{d}s}(1-\mathrm{e}^{-\omega^-\int_{t-T_D}^{t}\Xi\mathrm{d}s}) + p^+\mathrm{e}^{-\omega^+\int_0^{t-T_D}\Xi\mathrm{d}s}(1-\mathrm{e}^{-\omega^+\int_{t-T_D}^{t}\Xi\mathrm{d}s})\right)/ \\
&\left(p^-\mathrm{e}^{-\omega^-\int_0^{t-T_D}\Xi\mathrm{d}s} + p^+\mathrm{e}^{-\omega^+\int_0^{t-T_D}\Xi\mathrm{d}s}\right)
\end{aligned}
$$

$$I(t) = \Big(p^- \omega^- \Xi(t) \mathrm{e}^{-\omega^- \int_0^t \Xi \mathrm{d}s} + p^+ \omega^+ \Xi(t) \mathrm{e}^{-\omega^+ \int_0^t \Xi \mathrm{d}s}\Big) / \Big(p^- \mathrm{e}^{-\omega^- \int_0^t \Xi \mathrm{d}s} + p^+ \mathrm{e}^{-\omega^+ \int_0^t \Xi \mathrm{d}s}\Big)$$

General Inequalities

For arbitrary $0 < \tilde{t} < t$,

$$\begin{aligned} &\Pr(\mathcal{A}(t-\tilde{t},t)|\mathcal{A}(t-\tilde{t},\infty)) \\ &= \int (1 - \mathrm{e}^{-w \int_{t-\tilde{t}}^t \Xi(s) \mathrm{d}s}) \mathrm{d}g_\omega(w|\mathcal{A}(t-\tilde{t},\infty)) \end{aligned} \tag{32}$$

is, for each t, the integral of a *concave function* $C(\omega) = 1 - \mathrm{e}^{-\omega \int_{t-\tilde{t}}^t \Xi(s) \mathrm{d}s}$ over a probability distribution. Thus, it is generally true, by Jensen's Inequality [20], that

$$\Pr(\mathcal{A}(t-\tilde{t},t)|\mathcal{A}(t-\tilde{t},\infty)) \leq 1 - \mathrm{e}^{-\mathbb{E}[\omega] \int_{t-\tilde{t}}^t \Xi(s) \mathrm{d}s}. \tag{33}$$

A consequence of (33) is that for an arbitrary distribution g_ω within a population, the prevalence and incidence (for finite time steps) of AD will not exceed the prevalence and incidence in a population where every individual has the same value of ω_0. The same argument also shows that for an arbitrary distribution g_U within the population and with γ and t fixed, the average healthy neuronal density $V(t)$ will be at least as large as the healthy neuronal density in a population where every individual has the same value of U_0. The inequality is reversed because the exponential decay for V is a convex function.

Stochastic Simulation Details

The pseudo-code for our stochastic simulations can be summarized below. We used $M = 400$, $N = 40,000$, $t\_end = 90$ yr, $dt = 0.25$ yr. Note that for an individual, $U(t) = U_0 \Xi(t)$, where U_0 may be fixed or chosen from a distribution and $\Xi(t)$ may be constant (in the static model) or age-dependent (in the dynamic model).

```
for i = 1 to M: # number of simulations M
  t = 0

  for j = 1 to N: # number of people N
    # pick U0 from sample distribution
    choose value U0 ~ g(u)
    initialize person with U0
    add to population

  while t < t_end:
    for j = 1 to N:
      if person j has had AD more than T_D:
        die
        continue
      endif

     # generate exponential distribution
     # with hazard function
```

```
      choose I ~ Exp(gamma*U(t))
       if I < dt:
         person j gets AD
       endif

       t <- t + dt
   endwhile
 endfor
```

Additional Sensitivity Analysis Figures

Additional figures to illustrate the sensitivity analysis are provided in Figs C-E.

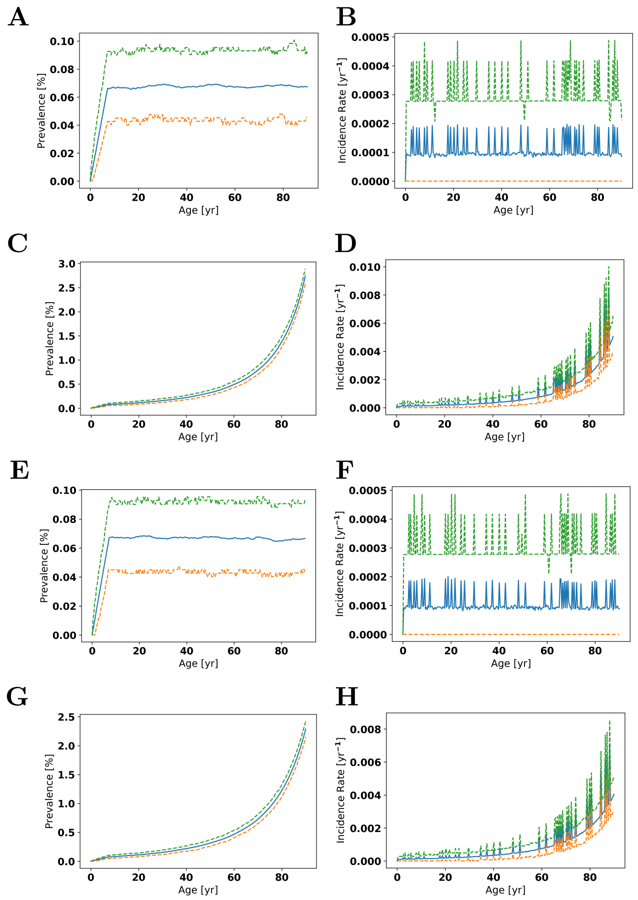

Fig C. Confidence Windows. The solid line is the simulation mean and the dashed lines represent the boundaries of the 95% confidence window. *A/B:* with U_0 fixed and static; *C/D:* with U_0 fixed and dynamic; *E/F:* with U_0 from example distribution and static; *G/H:* with U_0 from example distribution and dynamic.

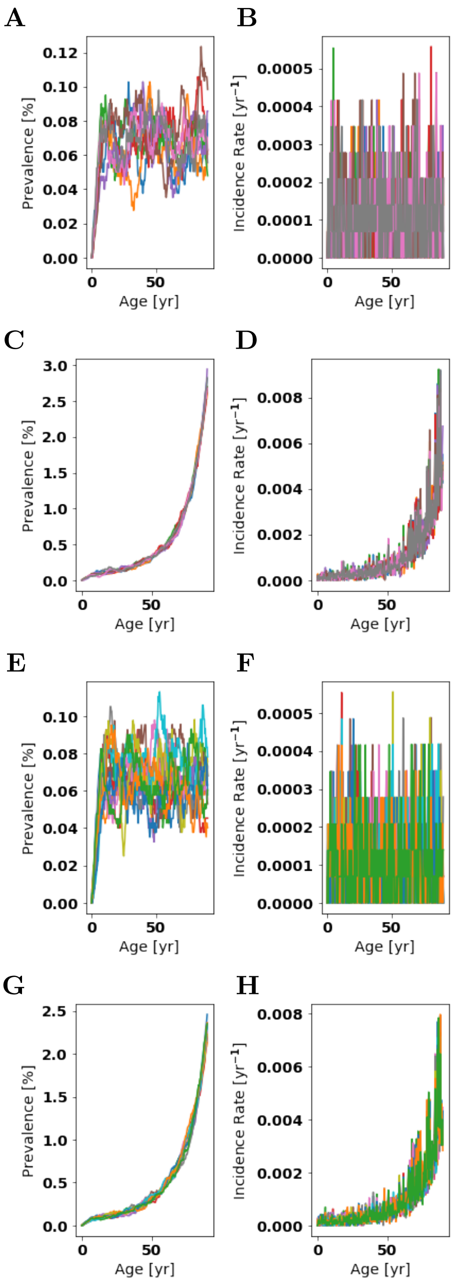

Fig D. Stochastic Trajectories. A few random trajectories of prevalence and incidence for the entire population. *A/B:* with U_0 fixed and static; *C/D:* with U_0 fixed and dynamic; *E/F:* with U_0 from the example distribution and static; *G/H:* from U_0 from example distribution and dynamic.

Time-Dependent Toxicity Rate σ

We assume that $\sigma(t) = \sigma_0 \times (1 + t/\lambda_\sigma)$. A first-degree Taylor expansion of $\omega(t)$ yields $\omega(t) = 1 + 2t/\lambda_S + 2t/\lambda_\kappa + t/\lambda_\sigma$. If incidence doubling every 4.9 yr corresponds to a growth of $\exp(t/\lambda^*)$ then $\lambda_\sigma = (1/\lambda^* - 2/\lambda_S - 2/\lambda_\kappa)^{-1}$. This leads us to a value of

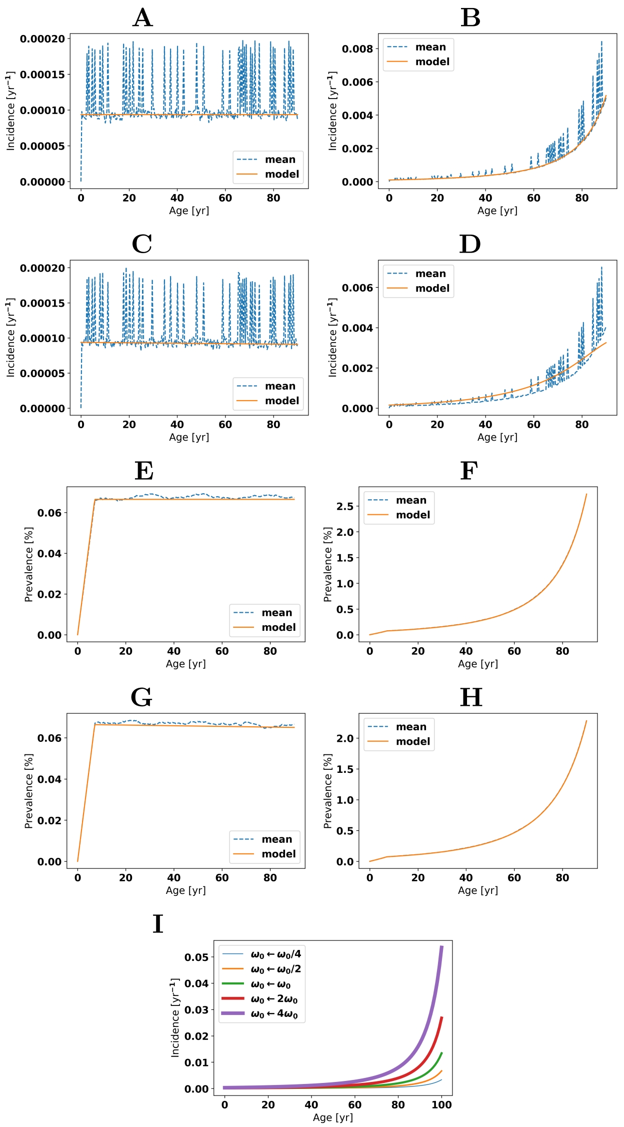

Fig E. Incidence Model. *A:* U_0 fixed and static; *B:* U_0 fixed and dynamic; *C*: U_0 from example distribution and static; *D:* U_0 from example distribution and dynamic. **Prevalence Model.** *E:* U_0 fixed and static; *F:* U_0 fixed and dynamic; *G*: U_0 from example distribution and static; *H:* U_0 from example distribution and dynamic. **Incidence variation with** ω_0**.** *I:* incidence for dynamic model with ω_0 varying.

$\lambda_\sigma = 9.01$ yr.

However, with all other constants fixed and if $\sigma_0 = \bar{\sigma}$, this will cause too much neuronal death and HV loss to match the observed data of HV loss at age 75. We therefore assume that $\sigma(t) = \sigma_0(1 + t/\lambda_\sigma)$, where $\sigma_0 = 0.107\bar{\sigma}$, so this new model with time-dependent σ has the same HV loss rate at age 75 as the old model. We choose γ again by matching the AD incidence rate at age 60 to find that $\gamma = 0.732$ instead of 0.601.

Plots of the clinical and model incidence and prevalence are provided in Fig F, in

addition to the HV for this alternative model. The agreement is better, and through a line of best fit, we find doubling times of 8.94 yr and 10.1 yr for the incidence and prevalence, respectively.

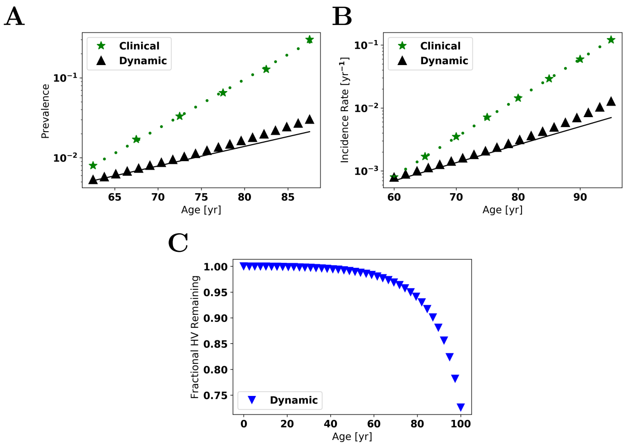

Fig F. Age-Dependent Toxicity. *A:* prevalence curve [21]. *B:* incidence curve [22]. *C:* HV curve.

References

1. Rinne H. The Hazard rate: Theory and inference (with supplementary MATLAB-Programs); 2014.

2. Lambert MP, Barlow A, Chromy BA, Edwards C, Freed R, Liosatos M, et al. Diffusible, nonfibrillar ligands derived from Aβ1–42 are potent central nervous system neurotoxins. Proceedings of the National Academy of Sciences. 1998;95(11):6448–6453.

3. Cizas P, Budvytyte R, Morkuniene R, Moldovan R, Broccio M, Lösche M, et al. Size-dependent neurotoxicity of β-amyloid oligomers. Archives of Biochemistry and Biophysics. 2010;496(2):84–92.

4. Norden R. A survey of maximum likelihood estimation. International Statistical Review/Revue Internationale de Statistique. 1972;p. 329–354.

5. Del Ferraro G, Moreno A, Min B, Morone F, Pérez-Ramírez Ú, Pérez-Cervera L, et al. Finding influential nodes for integration in brain networks using optimal percolation theory. Nature Communications. 2018;9(1):2274.

6. Raskatov JA. What Is the "Relevant" Amyloid β42 Concentration? ChemBioChem. 2019;.

7. Hansson O, Lehmann S, Otto M, Zetterberg H, Lewczuk P. Advantages and disadvantages of the use of the CSF Amyloid β (Aβ) 42/40 ratio in the diagnosis of Alzheimer's Disease. Alzheimer's research & therapy. 2019;11(1):1–15.

8. Oyama F, Cairns NJ, Shimada H, Oyama R, Titani K, Ihara Y. Down's syndrome: Up-regulation of β-amyloid protein precursor and τ mRNAs and their defective coordination. Journal of neurochemistry. 1994;62(3):1062–1066.

9. Rumble B, Retallack R, Hilbich C, Simms G, Multhaup G, Martins R, et al. Amyloid A4 protein and its precursor in Down's syndrome and Alzheimer's disease. New England Journal of Medicine. 1989;320(22):1446–1452.

10. Fukumoto H, Rosene DL, Moss MB, Raju S, Hyman BT, Irizarry MC. β-secretase activity increases with aging in human, monkey, and mouse brain. The American Journal of Pathology. 2004;164(2):719–725.

11. Nistor M, Don M, Parekh M, Sarsoza F, Goodus M, Lopez G, et al. Alpha-and beta-secretase activity as a function of age and beta-amyloid in Down syndrome and normal brain. Neurobiology of Aging. 2007;28(10):1493–1506.

12. Fukumoto H, Cheung BS, Hyman BT, Irizarry MC. β-Secretase protein and activity are increased in the neocortex in Alzheimer disease. Archives of Neurology. 2002;59(9):1381–1389.

13. Patterson BW, Elbert DL, Mawuenyega KG, Kasten T, Ovod V, Ma S, et al. Age and amyloid effects on human central nervous system amyloid-beta kinetics. Annals of Neurology. 2015;78(3):439–453.

14. Lue LF, Kuo YM, Roher AE, Brachova L, Shen Y, Sue L, et al. Soluble amyloid β peptide concentration as a predictor of synaptic change in Alzheimer's disease. The American Journal of Pathology. 1999;155(3):853–862.

15. Garai K, Frieden C. Quantitative analysis of the time course of Aβ oligomerization and subsequent growth steps using tetramethylrhodamine-labeled Aβ. Proceedings of the National Academy of Sciences. 2013;110(9):3321–3326.

16. Nag S, Sarkar B, Bandyopadhyay A, Sahoo B, Sreenivasan VK, Kombrabail M, et al. Nature of the amyloid-β monomer and the monomer-oligomer equilibrium. Journal of Biological Chemistry. 2011;286(16):13827–13833.

17. Murphy RM, Pallitto MM. Probing the kinetics of β-amyloid self-association. Journal of Structural Biology. 2000;130(2-3):109–122.

18. Mériaux S, Conti A, Larrat B. Assessing diffusion in the extra-cellular space of brain tissue by dynamic MRI mapping of contrast agent concentrations. Frontiers in Physics. 2018;6:38.

19. Jin M, Shepardson N, Yang T, Chen G, Walsh D, Selkoe DJ. Soluble amyloid β-protein dimers isolated from Alzheimer cortex directly induce Tau hyperphosphorylation and neuritic degeneration. Proceedings of the National Academy of Sciences. 2011;108(14):5819–5824.

20. Sturm KT. Probability measures on metric spaces of nonpositive. Heat Kernels and Analysis on Manifolds, Graphs, and Metric Spaces: Lecture Notes from a Quarter Program on Heat Kernels, Random Walks, and Analysis on Manifolds and Graphs: April 16-July 13, 2002, Emile Borel Centre of the Henri Poincaré Institute, Paris, France. 2003;338:357.

21. Ferri CP, Prince M, Brayne C, Brodaty H, Fratiglioni L, Ganguli M, et al. Global prevalence of dementia: a Delphi consensus study. The Lancet. 2005;366(9503):2112–2117.

22. Desikan RS, Fan CC, Wang Y, Schork AJ, Cabral HJ, Cupples LA, et al. Genetic assessment of age-associated Alzheimer disease risk: Development and validation of a polygenic hazard score. PLoS medicine. 2017;14(3):e1002258.
